# Microsatellite instability-related prognostic risk score (MSI-pRS) defines a subset of lung squamous cell carcinoma (LUSC) patients with genomic instability and poor clinical outcome

**DOI:** 10.3389/fgene.2023.1061002

**Published:** 2023-02-17

**Authors:** Zixin Hu, Zhening Liu, Jiabin Zheng, Yanmei Peng, Xingyu Lu, Jia Li, Kexin Tan, Huijuan Cui

**Affiliations:** ^1^ Beijing University of Chinese Medicine, Beijing, China; ^2^ Department of Oncology, China-Japan Friendship Hospital, Beijing, China; ^3^ Department of Oncology, Fangshan Hospital, Beijing, China

**Keywords:** lung squamous cell carcinoma, genomic instability, mismatch repair system, microsatellite instability, TP53, prognostic biomarkers

## Abstract

**Background:** Lung squamous cell carcinoma (LUSC) shares less typical onco-drivers and target resistance, but a high overall mutation rate and marked genomic complexity. Mismatch repair (MMR) deficiency leads to microsatellite instability (MSI) and genomic instability. MSI is not an ideal option for prognosis of LUSC, whereas its function deserves exploration.

**Method:** MSI status was classified by MMR proteins using unsupervised clustering in the TCGA–LUSC dataset. The MSI score of each sample was determined by gene set variation analysis. Intersections of the differential expression genes and differential methylation probes were classified into functional modules by weighted gene co-expression network analysis. Least absolute shrinkage and selection operator regression and stepwise gene selection were performed for model downscaling.

**Results:** Compared with the MSI-low (MSI-L) phenotype, MSI-high (MSI-H) displayed higher genomic instability. The MSI score was decreased from MSI-H to normal samples (MSI-H > MSI-L > normal). A total of 843 genes activated by hypomethylation and 430 genes silenced by hypermethylation in MSI-H tumors were classified into six functional modules. CCDC68, LYSMD1, RPS7, and CDK20 were used to construct MSI-related prognostic risk score (MSI-pRS). Low MSI-pRS was a protective prognostic factor in all cohorts (HR = 0.46, 0.47, 0.37; *p*-value = 7.57e-06, 0.009, 0.021). The model contains tumor stage, age, and MSI-pRS that showed good discrimination and calibration. Decision curve analyses indicated that microsatellite instability-related prognostic risk score added extra value to the prognosis. A low MSI-pRS was negatively correlated with genomic instability. LUSC with low MSI-pRS was associated with increased genomic instability and cold immunophenotype.

**Conclusion:** MSI-pRS is a promising prognostic biomarker in LUSC as the substitute of MSI. Moreover, we first declared that LYSMD1 contributed to genomic instability of LUSC. Our findings provided new insights in the biomarker finder of LUSC.

## 1 Introduction

Lung squamous cell carcinoma (LUSC) comprises 20% of non-small cell lung cancer (NSCLC) cases (Santos and Rodriguez, 2022). Compared with lung adenocarcinoma (LUAD), targetable genetic aberrations are not typical and target therapy is not ideal in LUSC. Immune checkpoint inhibitor (ICI)-based combination regimens have been moved into the first-line option, which led to a landmark change in the treatment of LUSC (Santos and Rodriguez, 2022). In contrast to LUAD, for smoking or other chemical exposures, the molecular profile of all LUSC stages is characterized by highly heterogeneous malignancy, with high genomic instability contributing to the high tumor mutational burden (Heist et al., 2012; Chen et al., 2022).

Genomic instability is an enabling hallmark of tumorigenesis and is the consequence of the DNA damage repair (DDR) system deficiency. DDR deficiency results from mutations of large-scale upstream cancer suppressor genes, such as *TP53*, which is commonly mutated across pan-cancer and in at least 80% of LUSC cases, and is enhanced by the following clonal evolution of cells (Mandal et al., 2019). DDR defects conventionally lead to either chromosomal instability (CIN) or microsatellite instability (MSI). CIN describes a wide variety of chromosomal abnormalities, including chromosomal rearrangements, deletions, insertions, and amplifications. MSI deriving from deficiency of DNA mismatch repair (MMR) manifests as the insertion of a few base pairs or deletion mutations, specifically at a repetitive sequence during DNA replication and genetic recombination (Zhao et al., 2019).

MMR involves a series of proteins which act in the manner of homodimers. MutS homologs (MSH2, MSH3, and MSH6) are responsible for detecting, recognizing, and binding mismatch errors. MutL homologs (MLH1 and PMS2) participate in the excision and synthesis of corrected DNA bases. Repression of transcription or functional defects in one or more MMR enzymes results in a systemic MMR deficiency (MMR-d). Hypermethylation and deletion mutations of MMR genes, especially those of MLH1 and MSH2, lead to transcriptional silence accounting for the majority of MMR-d. Alternations of MSH6 and PMS2 contribute a part of the remainder (Xiao et al., 2014; Yanagawa et al., 2021).

In the absence of an efficient correction system, tumors with MMR-d backgrounds are particularly sensitive to DNA mismatch errors and manifest as the accumulation of mutations in brief repetitive DNA sequences (microsatellite sites), which is acknowledged as microsatellite instability-high (MSI-H). MSI-H occurs in about 10%–25% of colorectal cancers, in about 5%–20% gastric cancers, and in about 13%–30% of endometrial cancers. MSI-H is associated with *TP53* mutations and high tumor mutation burden (TMB), which leads to tumor immunogenicity and stimulates the host anti-tumor immune response, thereby being sensitive to immune checkpoint inhibitors (ICIs).

The prevalence of MSI-H in NSCLC is not as prevalent as in the previously described cancers with frequencies of 0.17%–0.8% (Warth et al., 2016; De Marchi et al., 2022). MSI-H NSCLC samples were frequently associated with heavy smoking history and tended to be LUSC or sharing squamous components (Woenckhaus et al., 2003). MSI may not act as the driver factor as that in the inherited cancer and tend to represent a type of genomic instability in lung cancer (Pastuszak-Lewandoska et al., 2016). The correlation of genome instability and the response to ICIs has been attached with great importance in NSCLC. A past pan-cancer study declared a positive association between the DDR-associated gene defect and the prevalence of programmed cell death-ligand 1 (PD-L1) in NSCLC. In addition, patients with a DDR defect acquired clinical benefit from ICIs with improved median progression-free survival (mPFS) and median overall survival (mOS) (Chae et al., 2019). Compared with LUAD, LUSC shares more complex genomic instability. It was reported that a direct relationship between DDR gene variants and T cell activation was observed in LUSC rather than in LUAD (Kim et al., 2020). Therefore, the MSI-H phenotype is presumably meaningful in LUSC. Distribution of MMR expression potentially reflects the *de novo* mechanism of genome instability formation; thus, it remains to be a potential indicator in NSCLC.

So far, the clinical implication of MSI in LUSC remains unclear. In this study, MSI-related prognostic risk score (MSI-pRS) was established by machine learning and bioinformatics methods. The MSI status of LUSC samples was distinguished based on the expression of MMR systems. According to the MSI status, differentially expressed genes (DEGs) were identified and then classified into the functional gene modules by weighted correlation network analysis (WGCNA). Ultimately, four key MSI-related prognostic genes, CCDC68, LYSMD1, RPS7, and CDK20, were screened and used to construct a new risk score model named MSI-pRS to predict LUSC. We further analyzed the genomic features, immune infiltration, and the association with driver genes. Internal and external dataset validations were used to further verify the MSI-pRS model.

## 2 Methods

### 2.1 Data collection and processing

TCGA–LUSC level 3 RNA-seq data (HTSeq-Counts) were directly downloaded using the GDC data transfer tool ( *https://portal.gdc.cancer.gov/* ). The TCGA cohort was randomly assigned into a training cohort and a validation cohort at a ratio of 3:2 using the *caTools* package. GSE73403 (Agilent-014850, whole human genome microarray 4x44K G4112F) was downloaded from Gene Expression Omnibus (GEO) datasets ( *https://www.ncbi.nlm.nih.gov/* ). GSE135222 was used for exploring the association between ICI response and gene expression (Ritchie et al., 2015). HTSeq-counts were transformed into log-2-transformed transcripts per kilo-base per million mapped reads (TPM). Gene length was calculated as the sum of lengths of the non-redundant exon. Agilent one-color microarray intensity data were read by the *“read.maimages”* function, background-correlated by *“backgroundCorrect”* function, and normalized by the *“normalizeBetweenArrays”* function. The processes described previously were all implemented in the *limma* package (Aryee et al., 2014).

TCGA–LUSC DNA methylation data (IDATs) were also downloaded and read by the *“read.metharray.exp”* function applied in minfi and filtered by the *“champ.filter”* function in the *ChAMP* R package (Wilkerson and Hayes, 2010; Tian et al., 2017). Particularly, poor performing probes with *p*-value above 0.05, belonging to a sex chromosome, known to have common SNPs at the CpG sites, or having been demonstrated to be mapped to multiple places in the genome were removed before differential methylation analysis. Normalization was performed by the BMIQ method with the *“champ.norm”* function (Chae et al., 2019). TCGA–LUSC minus germline somatic copy number alternations (CNAs) and merged somatic simple-nucleotide variation (sSNV) segmented data of the cohort were downloaded from GDAC Firehose (Broad Institute TCGA Genome Data Analysis Center, *https://gdac.broadinstitute.org/* ) for analysis of mutation status.

### 2.2 Unsupervised classification of TCGA–LUSC samples for MSI status

An unsupervised clustering algorithm was applied to classify the MSI status of TCGA–LUSC samples based on the expression of seven genes encoding MMR proteins (MSH2, MSH3, MSH6, MLH1, MLH3, PMS2, and PMS1). The median absolute deviation (MAD) of the data matrix was used for further cluster analysis. 1,000 time repetitions were applied for guaranteeing the stability of classification. The agglomerative hierarchical clustering algorithm was based upon Pearson’s correlation distance. The highest cluster group was set as 6 (*k* = 6). The heatmap of consensus matrices, cluster-consensus plot, and item-consensus plot were used for defining the ultimate MSI clusters by taking the stability and purity of clusters into consideration (Hänzelmann et al., 2013). The aforementioned steps were carried out using the *ConsensusClusterPlus* package.

Gene set variation analysis (GSVA) was performed to derive the MSI score based on the MMR system gene set that contained the seven genes to identify the MSI status of each sample (Chalmers et al., 2017). Genomic instability of different groups based on MSI status was characterized and compared by measuring TMB, mutant-allele tumor heterogeneity (MATH), DNA ploidy status, and aneuploidy score. TMB was calculated as the rate of somatic non-synonymous mutations per megabase of sequenced DNA. The exome size was estimated as 38 Mb (Mayakonda et al., 2018). To evaluate tumor genomic heterogeneity, MATH was calculated as the MAD and the median of variant allele frequencies of non-synonymous variants using the *“inferHet*e*rogeneity”* implemented in *maftools* (Carter et al., 2012). DNA content is the main biologic index of tumor multiplication potentiality. Ploidy reflects the actual DNA content of cancer cells (Taylor et al., 2018). Aneuploidy reflects the imbalance and complication of DNA replication. DNA ploidy calculated using the Absolute algorithm and the aneuploidy score of TCGA–LUSC samples was directly downloaded from *https://gdc.cancer.gov/about-data/publications/panimmune* (Langfelder and Horvath, 2008).

### 2.3 Identification of MSI-related genes regulated by DNA methylation

DNA methylation is the critical epigenetical mechanism of regulating MSI through transcriptionally silenced or activated hub gene expression in the MSI-related signaling by hyper- or hypo-DNA methylation of gene promoters, including TSS200, TSS1500, 1stexon, and 5′UTR. We further obtained the MSI-related genes regulated by DNA methylation in the following three steps. First, differentially expressed genes (DEGs) were identified from the intersection of results calculated by two methods based on *limma* and DESeq2. Second, similar ways were used to obtain different methylation probes (DMPs) using *ChAMP* and *minfi* packages (Pastuszak-Lewandoska et al., 2016; Chae et al., 2019). Third, the overlapping of genes targeted by DMPs in promoters and DEGs was obtained. Genes with the reverse methylation and expression status were identified as MSI-related, which were regulated by DNA methylation and used for further prognostic analysis.

Gene annotation was based on Homo. sapiens GRCh38.p13 GFF3 (v35) file (GENCODE website, *https://www.gencodegenes.org/* ). The ensemble ID was converted into a gene symbol. Genes with duplicate annotation were represented by genes on the minor chromosomes. The threshold of DEGs and DMPs was defined as |logFC| > 0 and *p*-value <0.05.

### 2.4 Functional classification of epigenetically regulated MSI-related genes

MSI-related genes regulated by DNA methylation were classified into functional modules by gene co-expression networks using the *WGCNA* R package (Kuleshov et al., 2016). In this way, genes with similar patterns were grouped into the same module to realize feature dimension reduction. The soft thresholding power was set to 5 on the criterion of approximate scale-free topology by the *“pickSoftThreshold”* function. The weighted adjacency matrix was transformed into a topological overlap degree matrix (TOM). Hierarchical clustering was used to produce a hierarchical clustering tree of genes whose densely interconnected branches were highly co-expressed genes. Modules having shared similar expression profiles were simplified by a dynamic tree cut. Different colors represent different modules. We then quantified the associations of modules with an MSI phenotype to identify the MSI-related gene modules. Gene significance (GS) was defined as the absolute value of the correlation between the gene and the clinical phenotype. Module membership (MM) was defined as the correlation between the summary profile of the module and gene expression. The biological functions of gene modules were characterized by gene ontology (GO) using *EnrichR* (Gu et al., 2022).

### 2.5 Development of the MSI-related prognostic risk score (MSI-pRS)

The least absolute shrinkage and selection operator (LASSO) regression was performed using the *glmnet* R package for downscaling prognostic genes (Chen et al., 2021). Particularly, LASSO regression analyses were applied to the genes included in the functional modules classified in the 2.4 of the training cohort. The fitted lambda value for the model was screened by cross-validation. Prognostic genes derived from the modules were integrated for further stepwise variable selection procedure based on the multivariate Cox model to construct the ultimate MSI-pRS using the *My.stepwise* R package.

The expression matrix of the selected genes for the model was extracted, and the MSI-PRS of each sample was calculated using the following formula:

$$MSI-pRS=\overset{n}{\underset{i=0}{\sum }}\exp_{ji}*{coef}_{j}.$$

The MSI-pRS of sample i was calculated as the expression of candidate gene j in sample i, weighted by the coefficient in the multivariate Cox regression model. All the samples were stratified into high- and low- MSI-pRS groups. The cutting points were selected by the *“surv-cutpoint”* function implemented in the *survminer* R package, according to Chen et al. (Blanche et al., 2013). All potential cutting points were repeatedly tested to find the maximum rank statistic to reduce the calculated batch effect. We divided the whole cohorts based on the MSI-pRS to further explore the prognostic value of the MSI-pRS for convenient routine use. The *“surv-cutpoint”* function was used to dichotomize the MSI-pRS, and the Kaplan–Meier method was used and log-rank tests between groups were performed. Hazard ratios (HRs) of the MSI-pRS were derived from univariate Cox regression. Subgroup analysis of MSI-pRS groups was performed to eliminate the interference of interactive variables. The multivariate Cox model fitting into age, stage, and MSI-pRS was applied in training, internal validation, and external validation cohorts. Receiver operating characteristic (ROC) curves at years 1, 3, and 5 of the model were drawn to evaluate the discriminative ability of the MSI-pRS using the *timeROC* R package (Vickers and Elkin, 2006). The efficiency of the MSI-pRS was assessed by comparing the decision curve analyses (DCA) and curves of models with or without the MSI-pRS group (Charoentong et al., 2017). Statistical analysis was conducted using R software (version 4, 4.1.2). Survival analyses were carried out using the survival R package, and the forest plots were pictured using the *forestplot* R package.

### 2.6 Functional enrichment analysis

Tumor-infiltrated cells were estimated by single-sample gene set enrichment analysis (ssGSEA) using the *GSVA* package (Hänzelmann et al., 2013). Transcriptional data of tumor-infiltrating cells used for functional analysis were derived from Charoentong et al. (Rooney et al., 2015). The positive immune regulators were defined as the collection of “effector” cells, active dendritic cells (aDCs), natural killer cells (NKs), and natural killer T cells (NKTs). Negative immune regulators were defined as the collection of regulatory T cells (Tregs) and myeloid-derived suppressor cells (MDSCs). The “effector” cells were defined as active T cells (aCD4+T and aCD8+T) and effector memory T cells (CD4+Tem and CD8+Tem). Cytolytic activity (CYT) was used for evaluating immune activity and calculated as the geometric mean of granzyme A (GZMA) and perforin (PRF1) expression levels as previously defined (Cancer Genome Atlas Research Network, 2012). Functional enrichment analysis between groups was realized by GSVA based on gene expression data matrices.

## 3 Results

### 3.1 MSI status and the genomic instability features of LUSC

**FIGURE 1:**
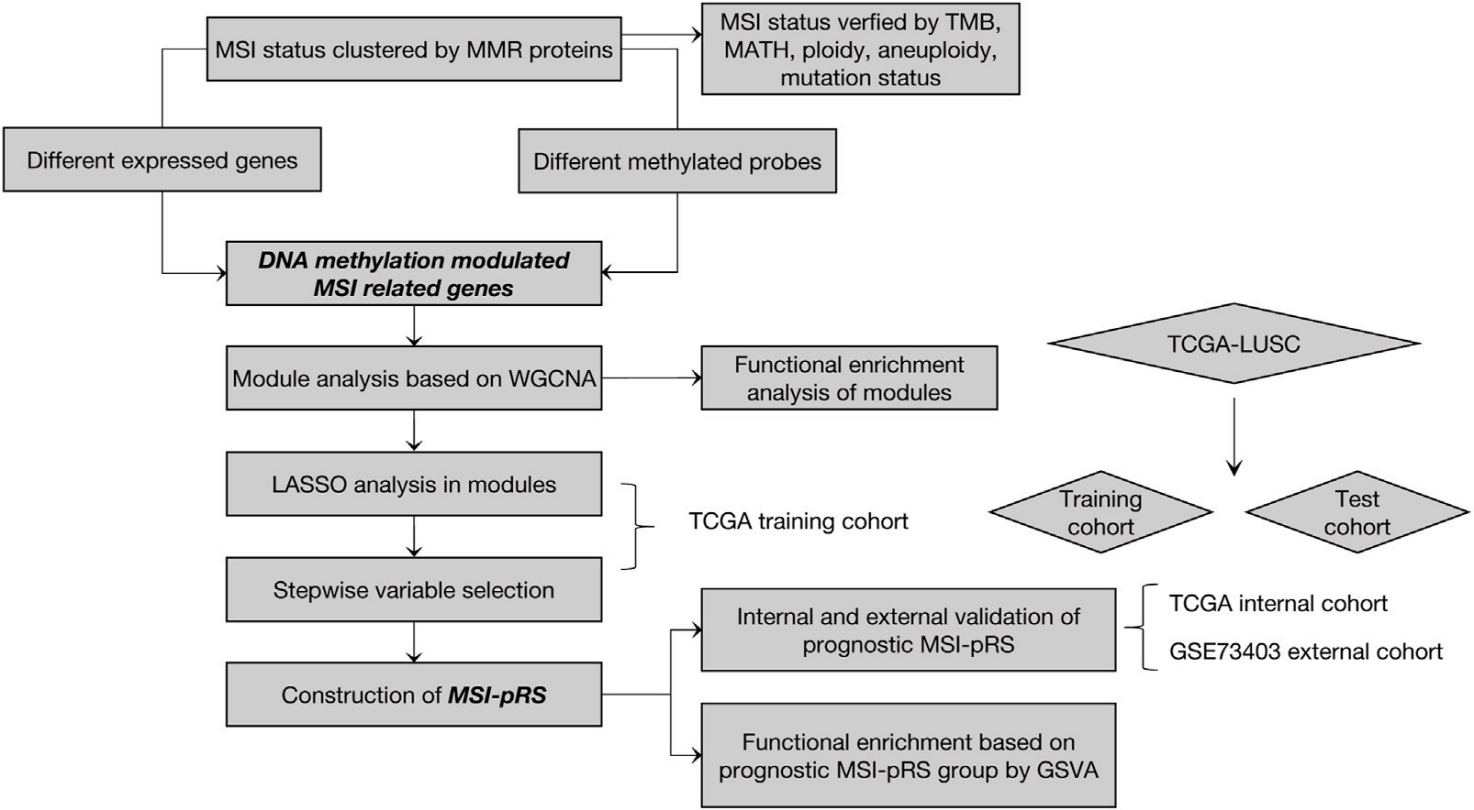
Process of MSI-related prognostic risk score (MSI-pRS) development.

The construction process of the MSI-pRS is shown in Figure 1. A total of 551 samples consisting of 502 tumor samples and 49 normal samples were contained in the TCGA–LUSC RNA-seq dataset. A total of 472 LUSC patients with clinical outcomes, transcriptomics, and mutation data were included for the analysis. The expression data of 49 normal samples were used for the control.

**FIGURE 2:**
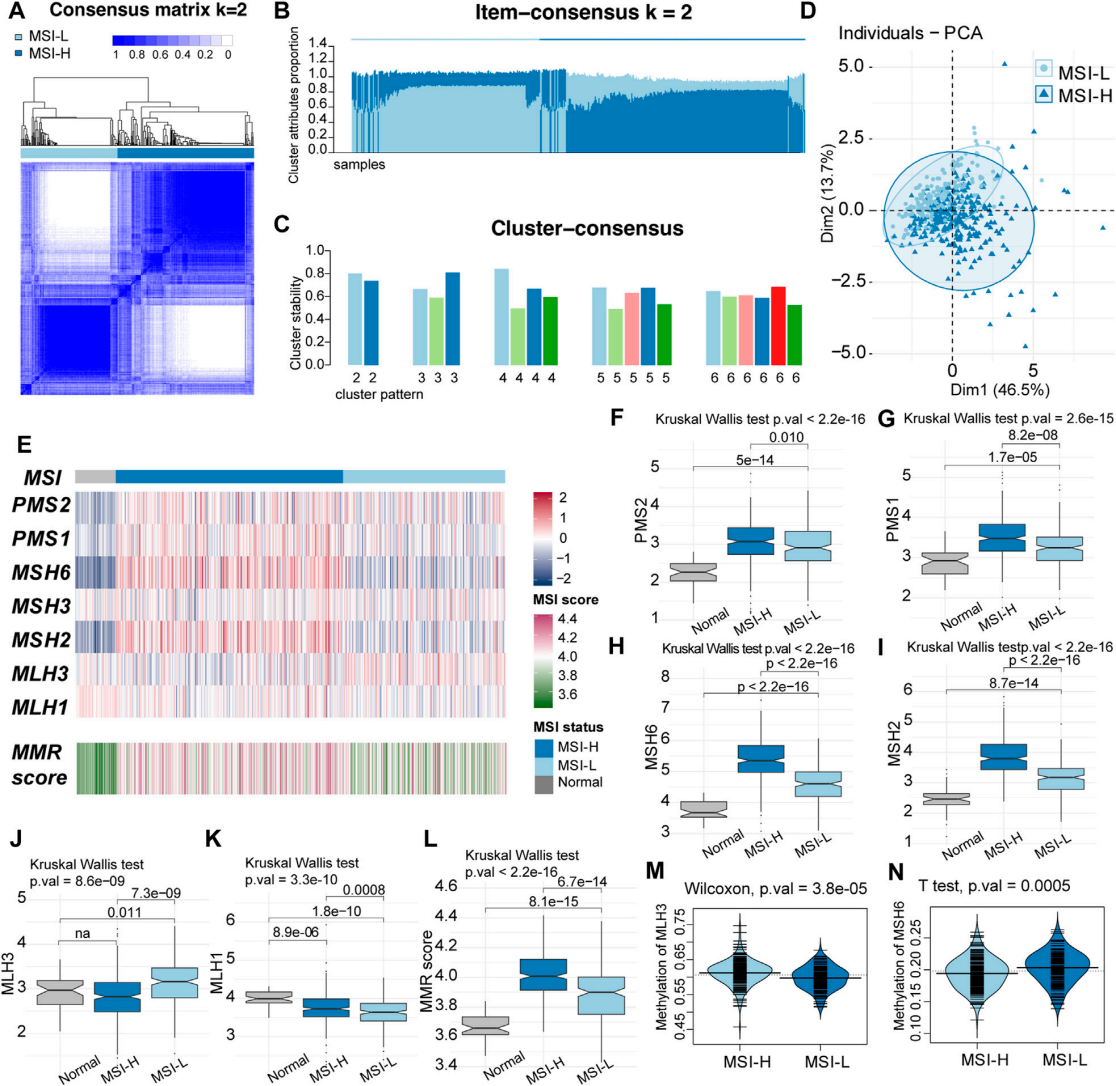
Two-group pattern of microsatellite instability (MSI) status by unsupervised classification in TCGA LUSC cohort. **(A)** Heatmap of consensus matrix when the classification pattern was two-group (k = 2). **(B)** Item consensus plot when k = 2 showed that the cluster pattern shared the acceptable purity in both groups. **(C)** Cluster consensus plot when k = 2 indicated the stability of the cluster patterns. **(D)** Principal component analysis (PCA) plot when k = 2 with 196 samples in MSI-L groups and 276 samples in the MSI-H group. **(E)** Comparation of the MMR proteins expression and the MSI score between MSI-H and MSI-L groups. MSI-H tended to had higher expression of MMR proteins and MSI score. **(F–K)** Comparation of MMR proteins among MSI-H, MSI-L and normal samples. **(L)** Boxplot of MMR score. MSI score was decreased from MSI-H samples to normal samples (MSI-H > MSI-L > normal). **(M–N)** Beanplot of methylation level of MLH3 and MSH6.

The MSI status was determined by the expression data of the MMR proteins in the following process: (Santos and Rodriguez, 2022) the relative MSI status in the TCGA–LUSC cohort was identified by consensus clustering using k-means as the base method (Chen et al., 2022). The MSI score of each sample was calculated by GSVA, according to MMR proteins. We tested clustering for *K* = 2–6 and chose the optimal number of subgroups using consensus matrices. According to the item-consensus plot and cluster-consensus plot, *K* = 2 showed crisp clusters with acceptable stability and purity in both groups (Figures 2A–C). The patients were divided into two robust groups with low MSI (MSI-L) containing 196 samples and high MSI (MSI-H) containing 276 samples.

The principal component analysis (PCA) plot preliminarily showed that the two groups had some difference in the expression of MMR proteins that somehow overlapped (Figure 2D). Particularly, the expression of MMR proteins and MSI scores were compared among normal samples, MSI-H, and MSI-L LUSC samples. Among the seven MMR proteins, PMS2, PMS1, MSH6, and MSH2 were significantly highest in the MSI-H group, while the expression level of the normal group was lowest (MSI-H > MSI-L > normal, Figures 2E–I). Expression of MLH3 was highest in MSI-L samples (Figures 2E, J), and that of MLH1 was highest in the normal samples and lowest in the MSI-H samples (Figures 2E, K). MSH3 was not significantly different among the groups. The MSI score was highest in the MSI-H group and lowest in the normal samples (MSI-H > MSI-L > normal, Figures 2E, L). The difference in the promoter methylation level of MMR genes was observed in MLH3, and MSH6 was consistent with the expression of the two proteins (Figures 2M, N). No significant difference in clinical characteristics was discovered between these two clusters of patients (Supplementary Table S1).

**FIGURE 3:**
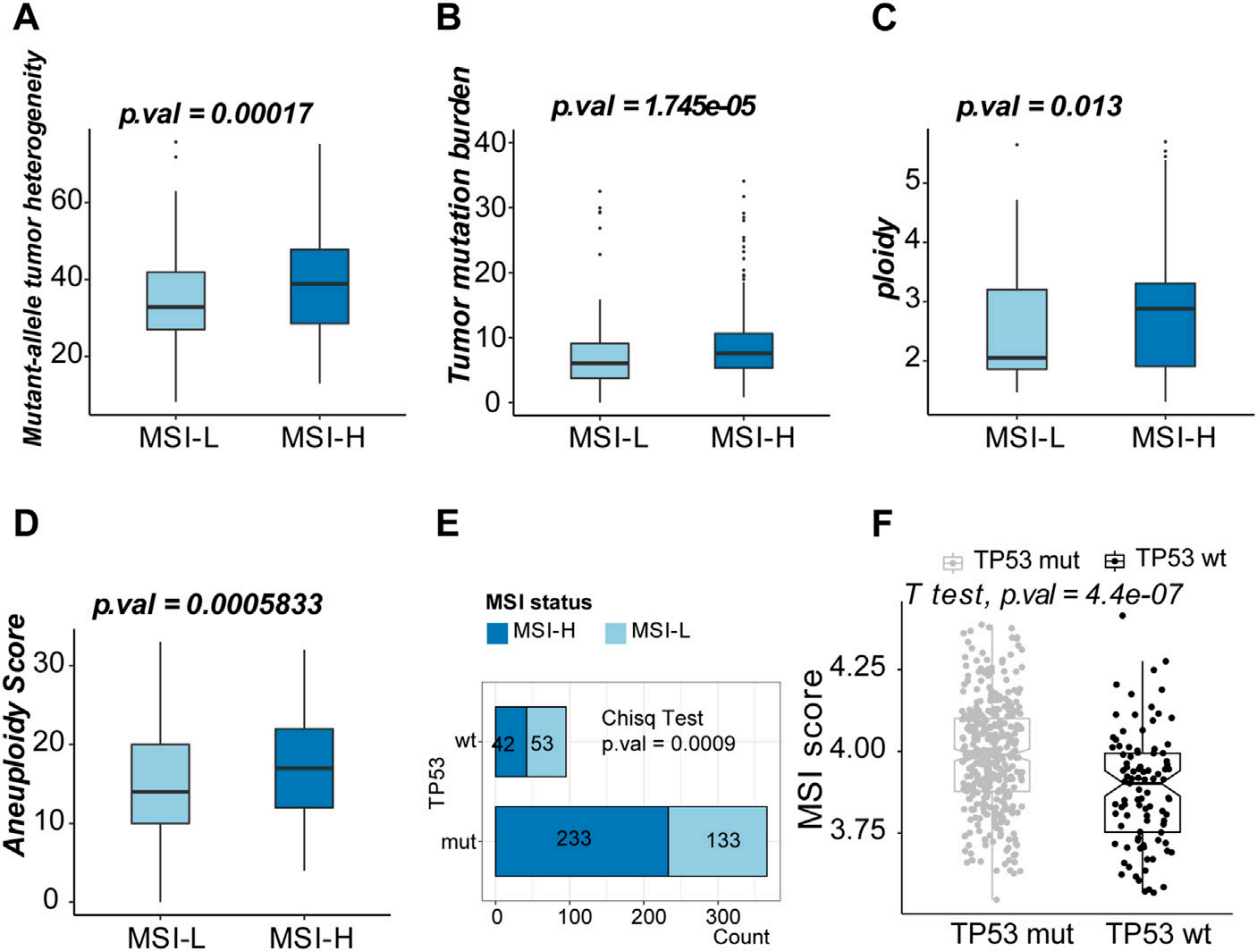
Exploration of the genomic features of MSI-H and MSI-L groups. **(A)** Boxplot of mutant–allele heterogeneity (MATH) (indicating intratumor heterogeneity). **(B)** Boxplot of tumor mutation burden (TMB). **(C)** Boxplot of ploidy (DNA content). **(D)** Boxplot of the aneuploidy score. **(E)** Comparison of the MSI scores between *TP53* mut and *TP53* wt groups. **(F)** Top 10 mutated genes in the MSI-L group. **(G)** Top ten mutated genes in the MSI-H group.

Genomic features between the two clusters were evaluated by TMB, tumor heterogeneity, DNA content, and aneuploidy status. The MSI-H group tended to obtain a subpopulation of heterogenous tumors with higher median MATH (Mann–Whitney *U*-test, 34.89 vs. 38.38, *p*-value = 0.002, Figure 3A). Higher TMB, DNA content (ploidy), and aneuploidy score were observed in the MSI-H group (Mann–Whitney *U*-test, 7.63/mb vs. 6.09/mb, *p*-value = 1.745e-05, Figure 3B; 2.88 vs. 2.05, *p*-value = 0.013; Figure 3C; 17 vs. 14, *p*-value = 0.001; Figure 3D). *TP53*, the alternation of which was the most universal mutation in LUSC patients, was more frequently mutated in the MSI-H group (chi-square test, *p*-value = 0.0009 Figure 3E). Moreover, the MSI score was higher in the patients of the *TP53* mut group (Student’s *t*-test, *p*-value = 4.4e-07, Figure 3F). To sum up, MSI-H in LUSC indicated a subgroup with higher genetic instability.

### 3.2 Identification of epigenetically regulated functional MSI-related genes

**FIGURE 4:**
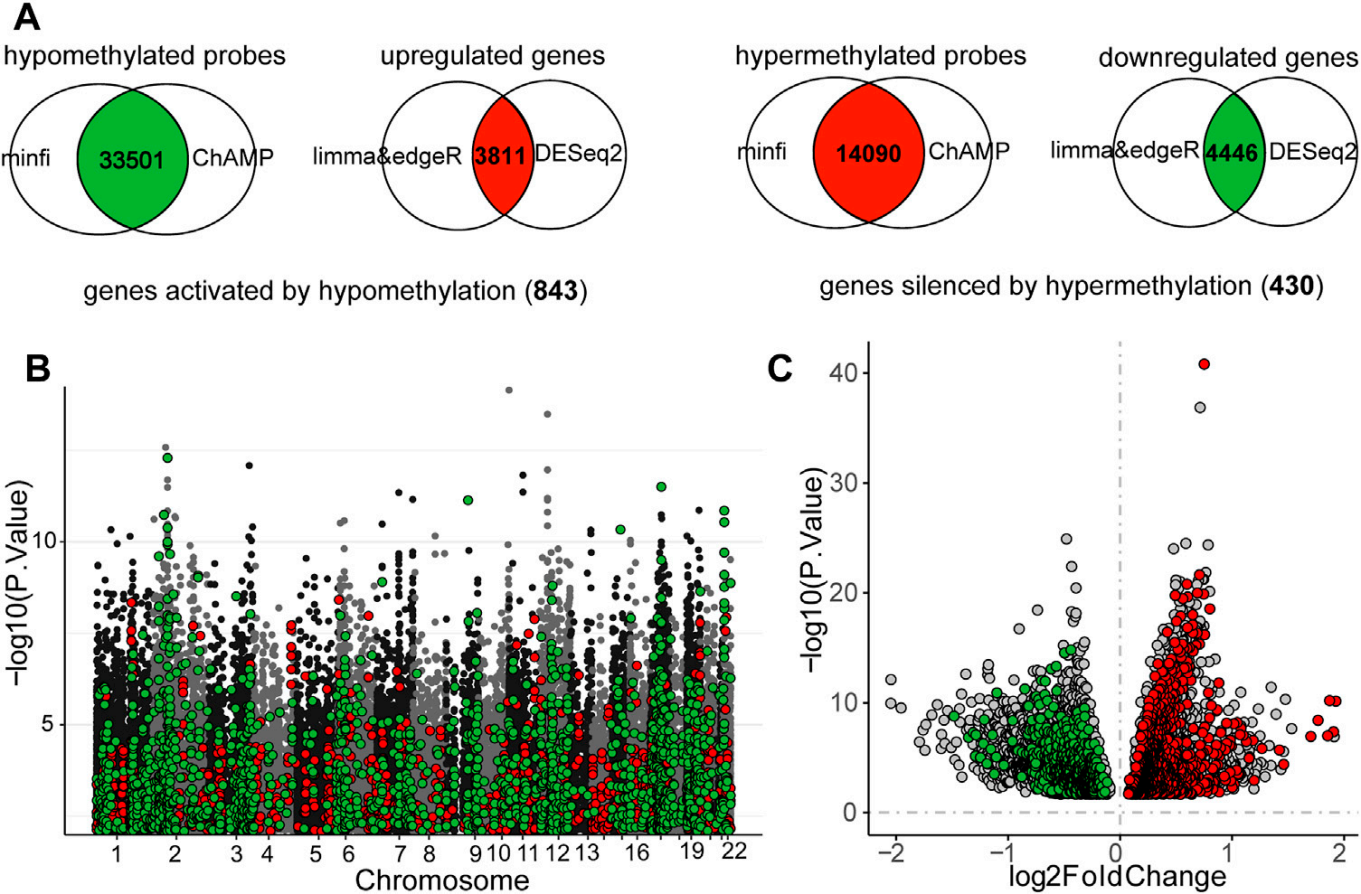
Intersection of the differentially expressed genes (DEGs) and different methylation probes (DMPs) to identify the DNA methylation regulated MSI-related genes. **(A)** Process of recognizing DEGs and DMPs. **(B)** Distribution of DNA methylation on chromosomes. MSI-related genes which were regulated by DNA methylation were highlighted. Red points represented probes that were hypermethylated in MSI-H, whereas green points indicated the probes were hypomethylated in MSI-H. **(C)** Volcano plot of DEGs between MSI-H and MSI-L groups. MSI-related genes which were regulated by DNA methylation were highlighted. Red points represented the upregulated MSI genes, and the green points represented the downregulated genes. Threshold of DEGs and DMPs was defined as: |logFC| > 0 and *p*-value <0.05.

There were 7,299 and 4,013 upregulated genes recognized by *limma* and *DESeq2* methods, respectively. The number of downregulated genes was 6,663 and 5,607. Ultimately, 3,811 DEGs were upregulated and 4,446 DEGs were downregulated in MSI-H samples. A total of 63,531 and 16,197 hypermethylated probes were calculated using *minfi* and *ChAMP*, while 63,547 and 45,387 probes were hypomethylated. The final DMPs were 14,090 hypermethylated probes and 33,501 hypomethylated probes in MSI-H samples. We eventually identified 843 genes activated by hypomethylation and 430 genes silenced by hypermethylation in MSI-H tumors (Supplemenatry Tables S2, S3; Figures 4A–C).

**FIGURE 5:**
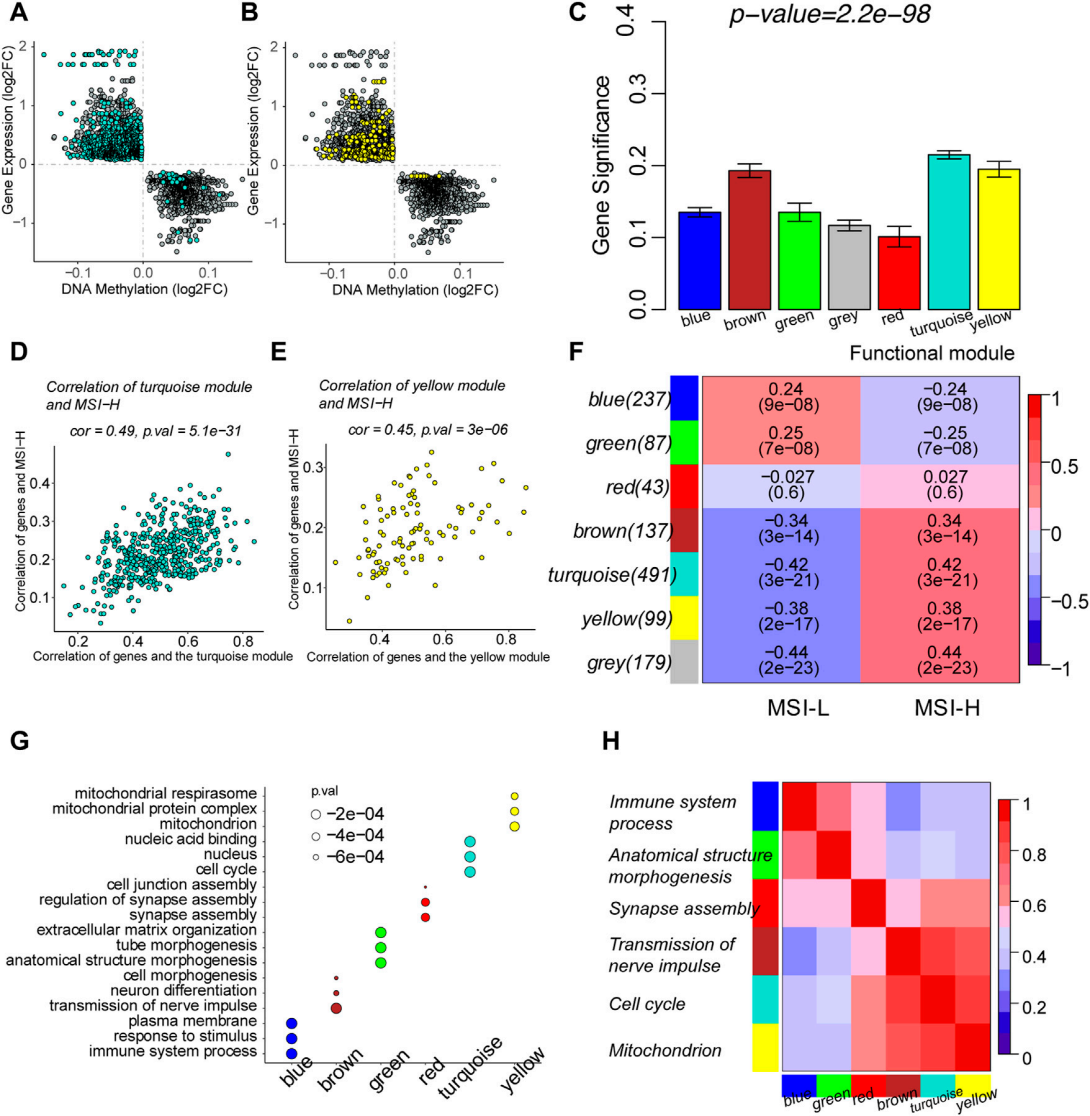
Identification of MSI-related functional gene modules by weighted correlation network analysis (WGCNA). **(A)** Scatterplots of the log2FC of DMPs and DEGs in the turquoise module. **(B)** Scatterplots of the log2FC of DMPs and DEGs in the yellow module. **(C)** Barplot of mean gene significance (GS) of each module. GS was defined as the absolute value of the correlation between the gene and the clinical phenotype. Since modules were clusters of functional genes, the mean GS of each module was correlated with GS of each gene in the module. **(D)** Scatterplots showed the correlation between GS and module membership (MM) in the turquoise module. MM was defined as the correlation between the summary profile of the module and the gene expression. **(E)** Scatterplots showed the correlation between GS and MM in the yellow module. **(F)** Relationship between functional modules and MSI phenotypes. Each row corresponded to a module containing functional genes, column to the MSI status. Each cell contains the corresponding correlation and *p*-value. The table is color-coded by correlation, according to the color legend. **(G)** Top three enriched biological processes of every module by functional enrichment of genes in each module. **(H)** Correlation among the functional modules. The top enriched biological process was used to represent the module in the y-axis.

We focused on the MSI-related genes which are epigenomically regulated by DNA methylation in the gene promoters and classified them into functional modules by gene co-expression networks. Six functional gene modules correlated with the MSI phenotype were identified. The gray module was the cluster of genes not related to any of the modules. Genes in the brown, turquoise, and yellow modules were more likely to be upregulated through DNA hypomethylation in the MSI-H group, whereas genes in blue and green modules tended to be downregulated by DNA hypermethylation in the MSI-H group (Figures 5A, B; Supplementary Figures S1A–D).

The correlation between modules and MSI was measured by MM and GS. MM indicated the correlation between the defined module and gene expression (Supplementary Table S4). GS represented the correlation between the gene and the clinical phenotype (Supplementary Table S4). The mean GS of each module was determined by the GS of each gene in the module (Figure 5C). The functional modules correlated with MSI were characterized by the high correlation of GS and MM of the genes in the module. Scatterplots of modules depicted the correlation between GS and MM (Figures 5D, E; Supplementary Figures S1E–H). GS and MM of genes in the yellow and turquoise modules are highly correlated with each other, illustrating that the genes in the modules are the central elements associated with the MSI. The association of the module and MSI is shown in the heatmap. The blue and green modules are negatively correlated with the MSI-H phenotype, while the brown, turquoise, and yellow modules were positively correlated with the MSI-H phenotype. A total of 179 genes were classified into the gray module and were thereby removed. Since there was no apparent correlation between the red module and MSI status, genes in the red module were waived for further prognostic analysis (Figure 5F). Functional enrichment was performed to illustrate the biological function of each module (Supplementary Table S5). The turquoise module represented a combination of genes that participated in cell cycle and DNA replication. The yellow module was crucially related to mitochondrion metabolism to provide energy for the biological process. The blue module gathered genes participating in the immune processes (Figures 4G, H).

### 3.3 Stepwise prognostic analysis of MSI-related genes and the MSI-pRS construction

The TCGA–LUSC cohort was randomly divided into training (*n* = 314) and validation (*n* = 158) cohorts for survival analysis. GSE73403 was used as the external validation cohort. The baseline clinicopathological features of the training cohort and validation cohorts are shown in Supplementary Table S6. MSI-related genes in the functional modules were selected to construct the MSI-pRS under the following two steps.

First, LASSO analyses based on the multivariate Cox model were carried out in the functional modules, except for the red module which is defined in the previously described process in the TCGA training cohort (Supplementary Figure S2). As a result, three genes,namely, ribosomal protein S7 (RPS7), cyclin-dependent kinase 20 (CDK20), and LysM domain containing 1 (LYSMD1) in the yellow module and three genes, namely, coiled-coil domain containing 68 (CCDC68), aldehyde dehydrogenase three family member B1 (ALDH3B1), and phosphodiesterase 1B (PDE1B) in the blue module were identified using a LASSO filtering (Supplementary Figures S2A–D). Genes in the turquoise, brown, and green modules failed to construct the model (Supplementary Figures S2E–J).

**FIGURE 6:**
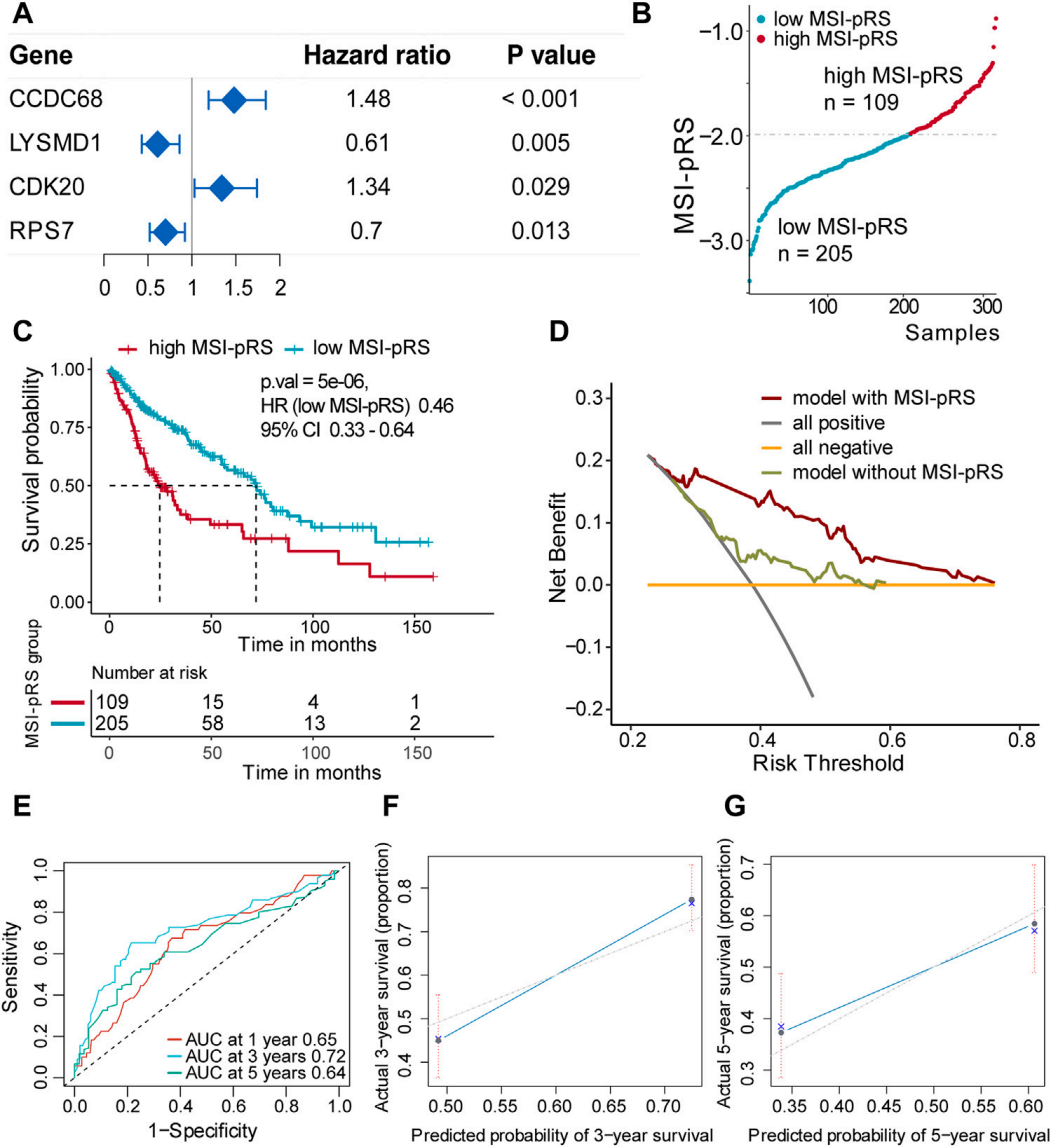
Construction of MSI-related prognostic risk score (MSI-pRS) in the TCGA training cohort. **(A)** Forest plot of prognostic genes used to construct MSI-PRS. Hazard ratio (HR) of each gene was derived from the univariate Cox regression of the four genes. **(B)** TCGA LUSC training cohort was divided into two groups according to the MSI-pRS. **(C)** Kaplan-Meier curves of the high MSI-pRS and low MSI-pRS groups in TCGA LUSC training cohort. The overall survival (OS) was used as the primary point. **(D)** Decision curves of the multivariate Cox model with or without MSI-pRS. The model with MSI-pRS declared a better performance than the model without the MSI-pRS group. **(E)** Receiver operating characteristic (ROC) curves at years 1, 3, and 5 of the model including age, tumor stage, and MSI-pRS group. **(F** and **G)** Calibration curves of the model, including age, tumor stage, and MSI-pRS group.

Second, genes selected in the previous step were brought into two-way stepwise regression to further simplify the model. Ultimately, four MSI-related genes, namely, CCDC68, LYSMD1, RPS7, and CDK20, were used to develop the MSI-pRS model (*p*-value = 1e-04, Table 1). Univariate Cox analysis indicated that the four genes were prognostic elements. LYSMD1 and RPS7 were protective factors, while CCDC68 and CDK20 were risk factors (Figure 6A). The coefficients obtained from the multivariate Cox regression were utilized as multiplicators, and the MSI-pRS was calculated as follows: MSI-PRS = CCDC68 expression level * 0.29 + LYSMD1 expression level * (−0.57) + RPS7 expression level * (−0.24) + CDK20 expression level * (0.43).

**TABLE 1:** Details of genes used for the construction of the MSI-related prognostic risk score (MSI-PRS).

Symbol	Protein	Location	MSI-H	Module	Genomic instability
CCDC68	Coiled-coil domain containing 68	18q21.2	Down	Blue	Suppressor
LYSMD1	LysM domain containing 1	1q21.3	Up	Yellow	Promoter
CDK20	Cyclin-dependent kinase 20	9q22.1	Up	Yellow	Promoter
RPS7	Ribosomal protein S7	2p25.3	Up	Yellow	Promoter

The MSI-pRS was a negative prognostic indicator according to univariate Cox regression in TCGA training, internal validation, and external validation cohorts (HR = 2.72, 95% CI 1.82–4.07, *p*-value = 1.22e-06; HR = 1.86 95% CI 1.01–3.43, *p*-value = 0.046; HR = 2.60, 95% CI 1.06–6.40, *p*-value = 0.037). The exploratory subgroup analyses in the three cohorts were performed (Supplementary Table S7). The low MSI-pRS was a protective factor in the male subgroup and the early stage (Stages I–II), which was consistent in all cohorts (Table 2).

**TABLE 2:** Subgroup analysis of the MSI-PRS in all LUSC cohorts.

	TCGA training cohort		TCGA validation cohort		GSE73403	
	HR (95% CI)	*p*-value	HR (95% CI)	*p*-value	HR (95% CI)	*p*-value
Male	2.79 (1.77–4.42)	1.15e-05	2.19 (1.13–4.24)	0.020	2.51 (1.02–6.15)	0.044
Early stage (stage I–II)	2.78 (1.73–4.47)	2.41e-05	2.75 (1.38–5.48)	0.004	7.41 (1.37–40.04)	0.020

**FIGURE 7:**
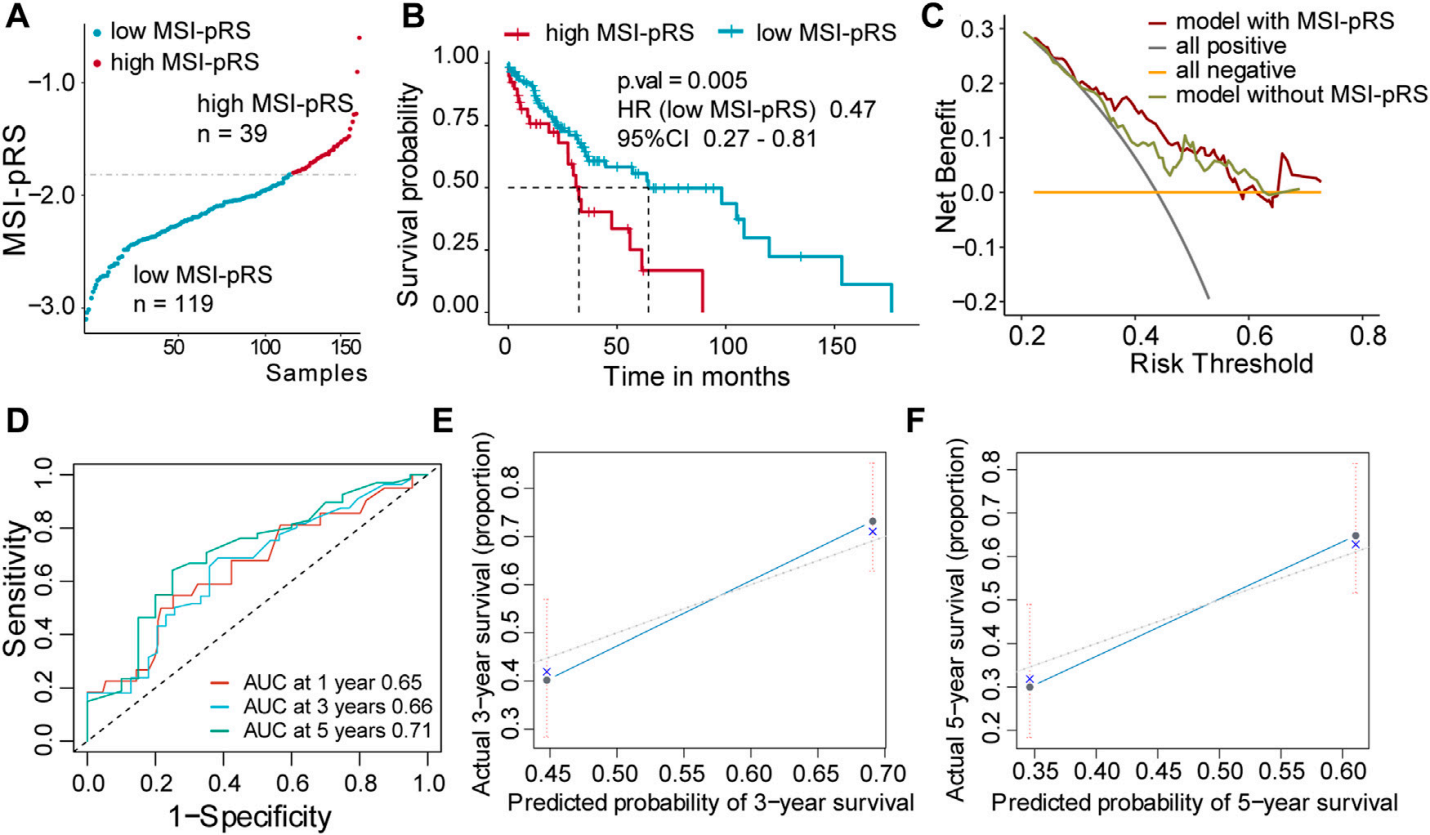
Validation of MSI-related prognostic risk score (MSI-pRS) in the internal test cohort (TCGA test cohort). **(A)** TCGA LUSC test cohort was divided into two groups according to MSI-pRS. **(B)** Kaplan-Meier curves of the high MSI-pRS and low MSI-pRS groups in TCGA LUSC test cohort. **(C)** Decision curves of the multivariate Cox model with or without MSI-PRS. The model with MSI-pRS declared a better performance than the model without the MSI-pRS group. **(D)** Receiver operating characteristic (ROC) curves at years 1, 3, and 5 of the model including age, tumor stage, and MSI-pRS group. **(E** and **F)** Calibration curves of the model, including age, tumor stage, and MSI-pRS group.

**FIGURE 8:**
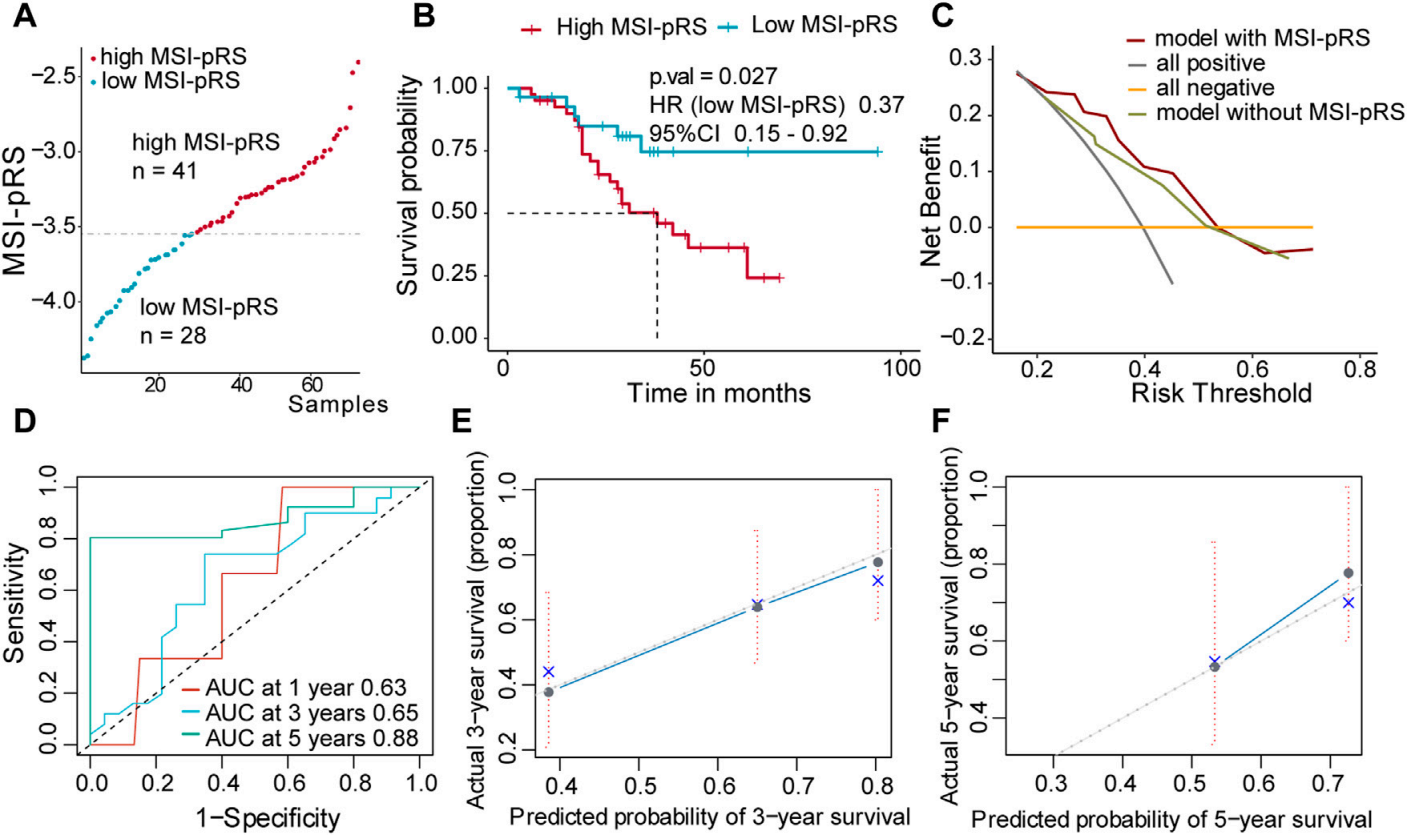
Validation of the MSI-related prognostic risk score (MSI-pRS) in the external test cohort (GSE73403 cohort). **(A)** All patients were divided into MSI-pRS high and MSI-pRS low groups. **(B)** Kaplan-Meier curves of the MSI-pRS high and MSI-pRS low groups in the external test cohort. **(C)** Decision curves of the multivariate Cox model with or without MSI-pRS. The model with the MSI-pRS declared a better performance than the model without the MSI-pRS group. **(D)** Receiver operating characteristic (ROC) curves at years 1, 3, and 5 of the model including age, tumor stage, and MSI-pRS group. **(E** and **F)** Calibration curves of the model including age, tumor stage, and MSI-pRS group.

Patients were assigned into two groups according to the MSI-pRS (Figures 6B, 7A, 8A). Low MSI-pRS was a protective prognostic factor in all cohorts (HR = 0.46, *p*-value = 7.57e-06, Figure 6C; HR = 0.47, *p*-value = 0.009; Figure 7B; HR = 0.37, *p*-value = 0.021; Figure 8B). The C-index of the univariate model of the MSI-pRS group was 0.72 (95% CI 0.64–0.80), 0.65 (95% CI 0.51–0.78), and 0.70 (95% CI 0.50–0.90) in the three cohorts. We then brought age, tumor stage, and MSI-pRS group into the multivariate Cox model to explore whether MSI-pRS added an incremental discriminative value to the clinical use. The model with the MSI-pRS was superior to the one without the MSI-pRS in all cohorts (*p*-value = 4.64−06, 0.008, 0.040). DCA curves of the two models demonstrated that the model with the MSI-pRS achieved better performance in all cohorts (Figures 6D, 7C, 8C).

The predictive accuracy of the MSI-pRS in the multivariate Cox model was evaluated by the time-dependent ROC and C-index. The C-index of the multivariate model was 0.64 (95% CI 0.59–0.69), 0.62 (95% CI 0.54–0.69), and 0.65 (95% CI 0.55–0.75) in the three cohorts. The area under the ROC curve (AUC) at 1 year, 3 year, and 5 year of the models with the MSI-pRS was 0.65, 0.72, and 0.64, respectively, in the TCGA training cohort; 0.65, 0.66, and 0.71 in TCGA validation cohort; 0.63, 0.65, and 0.88 in the external validation cohort (Figures 6E, 7D, 8D). The calibration curves showed that the model presents satisfied coherence between the actual survival and predicted survival rates (Figures 6F, G, 7E, F, 8E, F).

### 3.4 Correlation between the MSI-PRS and MSI status in LUSC

**FIGURE 9:**
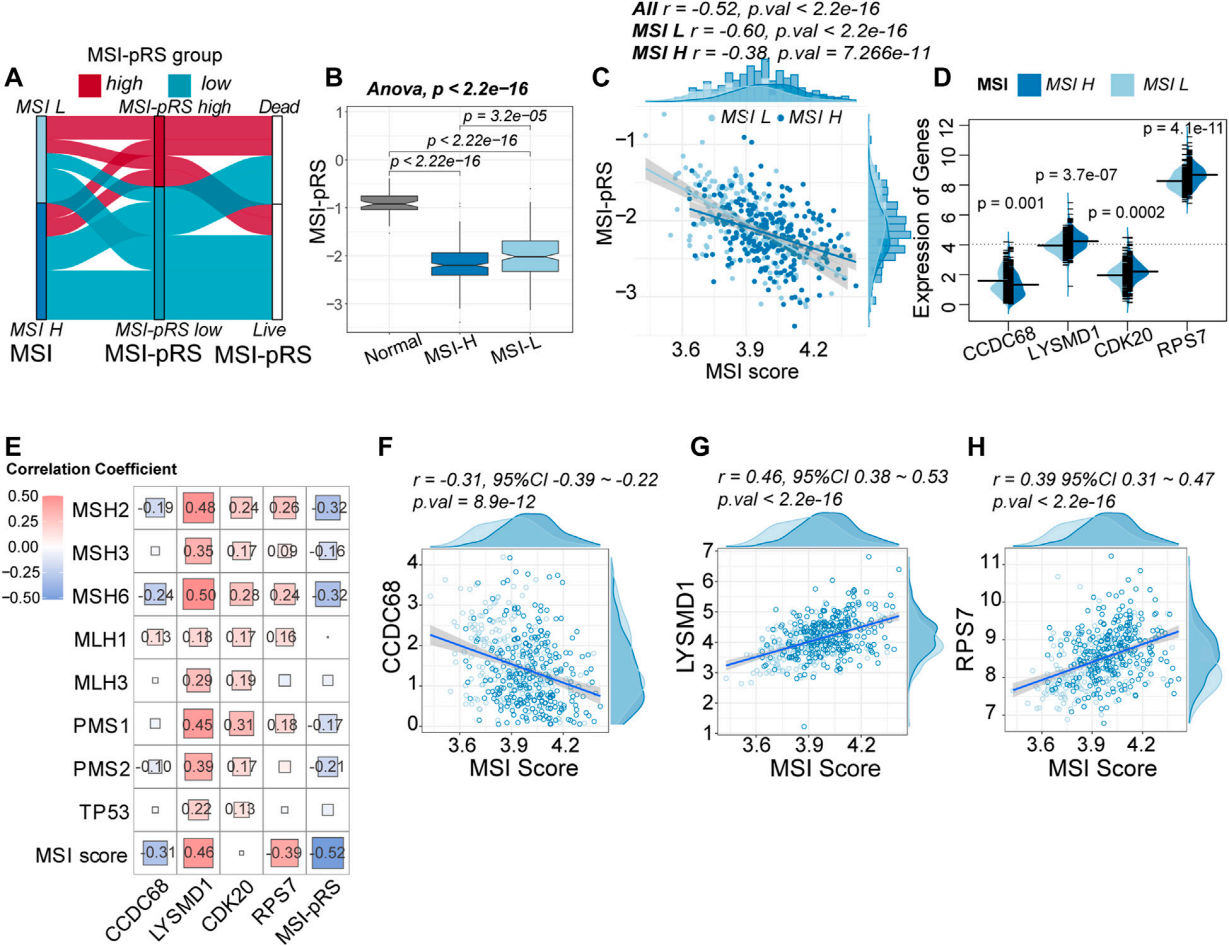
Relationship between the MSI-related prognostic risk score (MSI-pRS) and MSI status. **(A)** Flow diagram depicting the correlation among MSI status, MSI-pRS group, and clinical outcome in the TCGA LUSC training cohort. **(B)** Comparison of the MSI-pRS among normal samples, MSI-L samples, and MSI-H samples. MSI-pRS was decreased from the normal samples to the MSI-H samples (normal > MSI-L > MSI-H). **(C)** Correlation between MSI score and MSI-pRS. MSI-pRS was negatively correlated with MSI score. **(D)** Comparison of the four elements used to construct the MSI-pRS model between MSI-H and MSI-L groups. LYSMD1, CDK20, and RPS7 were higher in the MSI-H group, while CCDC68 was higher in the MSI-L group. **(E)** Relationship between MMR systems and MSI-pRS. **(F)** Correlation of CCDC68 and MSI scores. **(G)** Correlation of LYSMD1 and MSI scores. **(H)** Correlation of RPS7 and MSI scores.

Patients with MSI-H tended to have lower MSI-pRS (Student’s *t*-test, mean MSI-PRS: −2.18 vs. −2.019, *p*-value = 2.99e-05, Figure 9A; Supplementary Figure S3A). The MSI-pRS of normal samples was calculated for control. Compared with normal patients, LUSC samples had lower MSI-pRS (ANOVA test, *p*-value <2.2e-06, Figure 9B). The MSI-pRS was negatively correlated with MSI score. The correlation of the MSI-pRS and MSI score in the MSI-L group was higher than that of the MSI-H group (MSI-L: *r* = −0.60, 95% CI −0.68∼−0.50, *p*-value <2.2e-16; MSI-H: *r* = −0.38, 95% CI −0.48∼−0.27, *p*-value = 7.266e-11; Figure 9C).

The expression of the four genes used for constructing the MSI-PRS model was compared between the MSI-H and MSI-L group. Except for CCDC68, the other three genes were upregulated in the MSI-H group (Figure 9D). The correlation between MMR proteins and four genes used to construct the MSI-pRS was analyzed (Figure 9E; Supplementary Figure S3C, D). LYSMD1 was significantly positively related with MMR proteins, especially in the *TP53* wt cohort (Supplementary Figure S3C, D). The same trend was also observed in CDK20. Despite the positive correlation between CDK20 and MMR proteins, the correlation between CDK20 and MSI score was weak. LYSMD1 and RPS7 were positively correlated with MSI score, whereas CCDC68 was negatively correlated with MSI score (Figures 9F–H).

### 3.5 Low MSI-pRS related to *TP53* mutation and DNA hypomethylation of *TP53*

**FIGURE 10:**
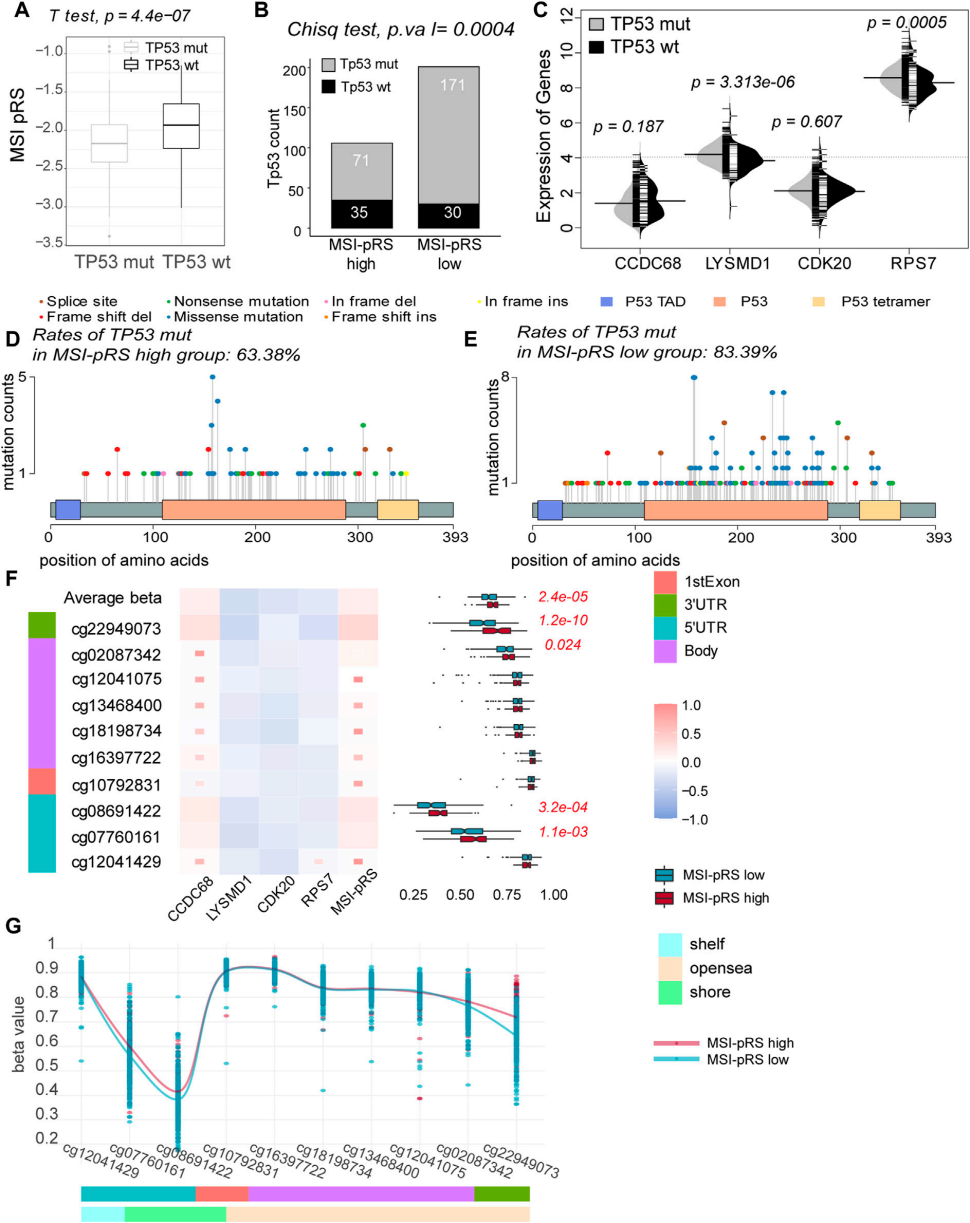
Relationship between the MSI-related prognostic risk score (MSI-pRS) and *TP53* status. **(A)** *TP53* mut had lower MSI-pRS than the TP53 wt. **(B)** Incidence of *TP53* mutations was more frequent in the low MSI-pRS group in the TCGA training cohort. **(C)** Comparison of the four elements used to construct the MSI-pRS model between *TP53* mut and *TP53* wt groups. LYSMD1 and RPS7 were higher in the *TP53* mut group. **(D)** Landscape of *TP53* mutations in the MSI-pRS high group. **(E)** Landscape of TP53 mutations in the MSI-pRS low group. **(F)** Correlation of the MSI-PRS and its elements with DNA methylation probes on *TP53* genes. MSI-pRS and CCDC68 were positively correlated with *TP53* DNA methylation, whereas LYSMD1, CDK20, and RPS7 were negatively correlated with *TP53* DNA methylation. **(G)** Comparison of DNA methylation on *TP53* genes between MSI-pRS high and low groups in TCGA LUSC cohort.

*TP53* mut tended to have lower MSI-pRS (Student’s *t*-test, *p*-value = 4.4e-07, Figure 10A). *TP53* had a higher mutation rate in the MSI-pRS low group in the training and internal validation cohorts (chi-square test; *p*-value = 0.0004, 0.008; Figure 10B; Supplementary Figure S4A). The comparison of four genes between the *TP53* mut and *TP53* wt groups was performed. The expression of LYSMD1 was significantly higher in the *TP53* mut group, whereas those of the other three genes showed no difference (Mann–Whitney’s *U*-test, *p*-value = 3.313e-06; Figure 10C).

Transactivation domains (TADs) mediate the transcriptional activity. A total of three and nine mutations on TADs were, respectively, observed in the MSI-pRS high and MSI-pRS low groups. There was no difference in the mutations of TADs in the two groups. The DNA-binding domain (DBD) enables p53 protein sequence-specific binding to DNA with a highly conserved structure. Mutations of *TP53* concentrated on the DBD. Missense mutation was the most common mutation type in both groups (chi-square test; MSI-pRS high: 54.1%, MSI-pRS low: 64.5%; *p*-value = 0.083; Figures 10D, E). The frequent mutations at six hotspots, i.e., codons R175, G245, R248, R249, R273, and R282, were 9.1% in the MSI-pRS high group while 16.4% in MSI-pRS low group (Chi-square test; *p*-value = 0.064; Figures 10D, E).

DNA hypomethylation commonly removes suppression of genes. The average beta value of DNA methylation on *TP53* genes was lower in the MSI-pRS low group (Mann–Whitney’s *U*-test, *p*-value = 2.4e-05), especially that on the promoters of *TP53* (cg12041429, cg07760161, cg08691422, and cg10792831, Mann–Whitney’s *U*-test, *p*-value = 2.0e-04, Figures 10F, G). The expression of *TP53* was not significantly different in the MSI-pRS high and -low groups (Mann–Whitney’s *U*-test; *p*-value = 0.151). MSI-PRS and CCDC68 were positively correlated with DNA methylation of *TP53*, while LYSMD1, CDK20, and RPS7 were negatively correlated with DNA methylation of *TP53*.

### 3.6 Low MSI-PRS associated with genomic instability

**FIGURE 11:**
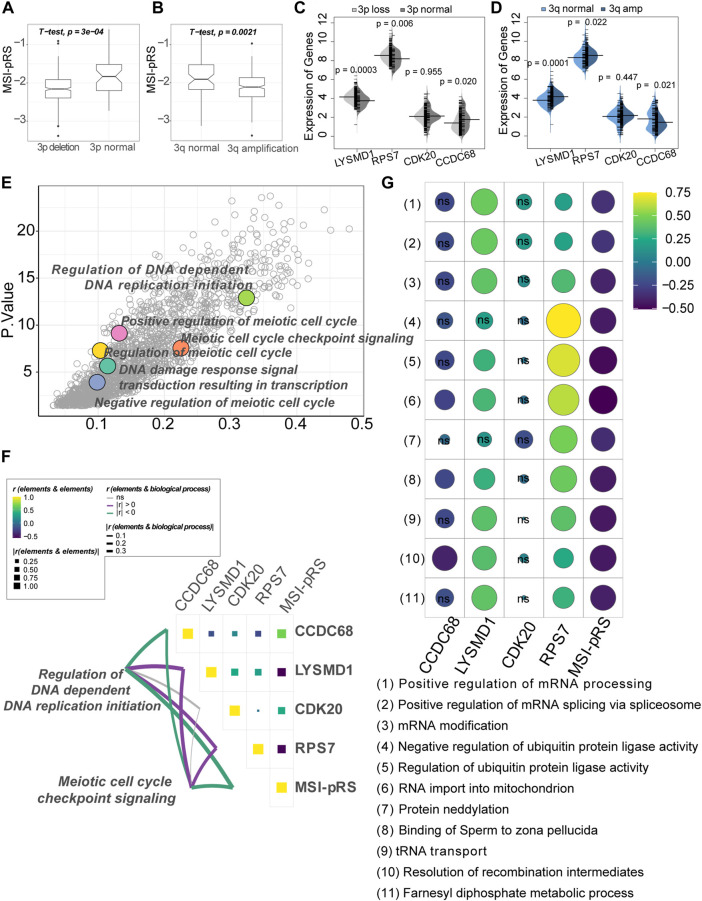
Genomic feature exploration of the MSI-related prognostic risk score (MSI-PRS). **(A)** MSI-pRS was lower in the chromosome 3p deletion LUSC. **(B)** MSI-pRS was lower in the chromosome 3q amplification LUSC. **(C)** Comparison of the four elements used to construct the MSI-pRS model between 3p loss and 3p normal groups. LYSMD1 and RPS7 were higher in the 3p loss group, while CCDC68 was lower in the 3p loss group. **(D)** Comparison of the four elements used to construct the MSI-pRS model between 3q amplification and 3q normal groups. LYSMD1 and RPS7 were higher in the 3q amplification group, while CCDC68 was lower in the 3q amplification group. **(E)** DNA replication, DNA damage response, and cell cycle checkpoint were upregulated in the MSI-pRS low group. Low MSI-pRS was associated with high genomic instability with active DNA damage repair response, whereas high MSI-pRS was *vice versa*. **(G)** Correlation of MSI-pRS and the transcriptional/ translational control associated biological processes. High MSI-pRS was negatively associated with transcriptional/translational control. LYSMD1 and RPS7 were positively correlated with the previously described processes. **(F)** Correlation of the MSI-pRS and its four elements. LYSMD1 and RPS7 were negatively correlated with the MSI-pRS, while CCDC68 and CDK20 were positively correlated with the MSI-pRS. Correlation of the MSI-pRS and four elements and DNA replication and cell cycle checkpoint processes. MSI-pRS and CCDC68 negatively related to the processes, whereas LYSMD1 and RPS7 were positively correlated.

Chromosome 3p alternations are an acknowledged feature of LUSC, with chromosome 3p loss and 3q amplification involved in the tumorigenesis of LUSC. The MSI-pRS was lower in the 3p deletion and 3q amplification group (Student’s *t*-test; 3p loss vs. 3p normal: −2.15 vs. −1.85, *p*-value = 0.0003; 3q amp vs. 3q normal: −2.11 vs. −1.89, *p*-value = 0.002; Figure 11A, B). The MSI-pRS low group had higher TMB, MATH, and aneuploidy scores (Mann–Whitney’s *U*-test; *p*-value = 0.0004, 0.019, 0.015; Supplementary Figures S4B–D). The MSI-pRS high group had higher subclonal genome fraction (Mann–Whitney’s *U*-test; *p*-value = 0.006; Supplementary Figures S4E). DNA content had no significant difference between MSI-pRS groups (Supplementary Figures S4F). LYSMD1 and RPS7 were higher in the 3p loss and 3q amplification groups than in the chromosome 3 normal samples, whereas CCDC68 was higher in the chromosome normal samples (Mann–Whitney’s *U*-test; Figures 11C, D).

Functional exploration was performed in the TCGA–LUSC cohort and GSE73403 validation cohort using the GO BP dataset by GSVA, according to the MSI-pRS group. Results obtained from the three cohorts (the training cohort, internal test cohort, and external test cohort) are intersected to obtain the final functional enrichment results. DNA-dependent DNA replication initiation and DNA damage response signal transduction resulting in transcription were upregulated in the MSI-pRS low group (Figure 11E). In particular, a number of meiotic cell cycle-related processes were upregulated in the MSI-pRS low group in all cohorts.

Biological processes that highly correlated with MSI-pRS and the expression of genes used to construct the MSI-pRS were explored. MSI-pRS was negatively correlated with meiotic cell cycle checkpoint signaling and DNA-dependent DNA replication initiation (Figure 11F; Supplementary Figure S5A). Moreover, MSI-pRS was negatively correlated with transcriptional and translational control, including mRNA and protein modification (Figure 11F; Supplementary Figures S5B).

Among the four genes comprising MSI-PRS, LYSMD1 and RPS7 were negatively related to DNA replication initiation and meiotic cell cycle checkpoint signaling and positively correlated with the transcriptional control (Figures 11F, G; Supplementary Figures S5B, C). LYSMD1 was highly correlated with the mRNA modification in the TCGA–LUSC cohort and the GSE73403 cohort, while RPS7 was tightly correlated with ubiquitin protein ligase activity in all cohorts (Figures 11F, G; Supplementary Figures S5B). CCDC68 was negatively correlated with the DNA replication initiation process and meiotic cell cycle checkpoint signaling but had a weak relationship with transcriptional and translational control processes. Although the expression of CDK20 was weakly correlated with all explored functional processes, it was the critical elements that composed the MSI-pRS (TCGA-LUSC: *r* = 0.38, *p*-value < 2.2e-16; GSE73403: *r* = 0.52, *p*-value = 4.2e-06; Figures 11F, G; Supplementary Figures S5A, B).

### 3.7 High MSI-pRS was characterized with an inflamed TME

**FIGURE 12:**
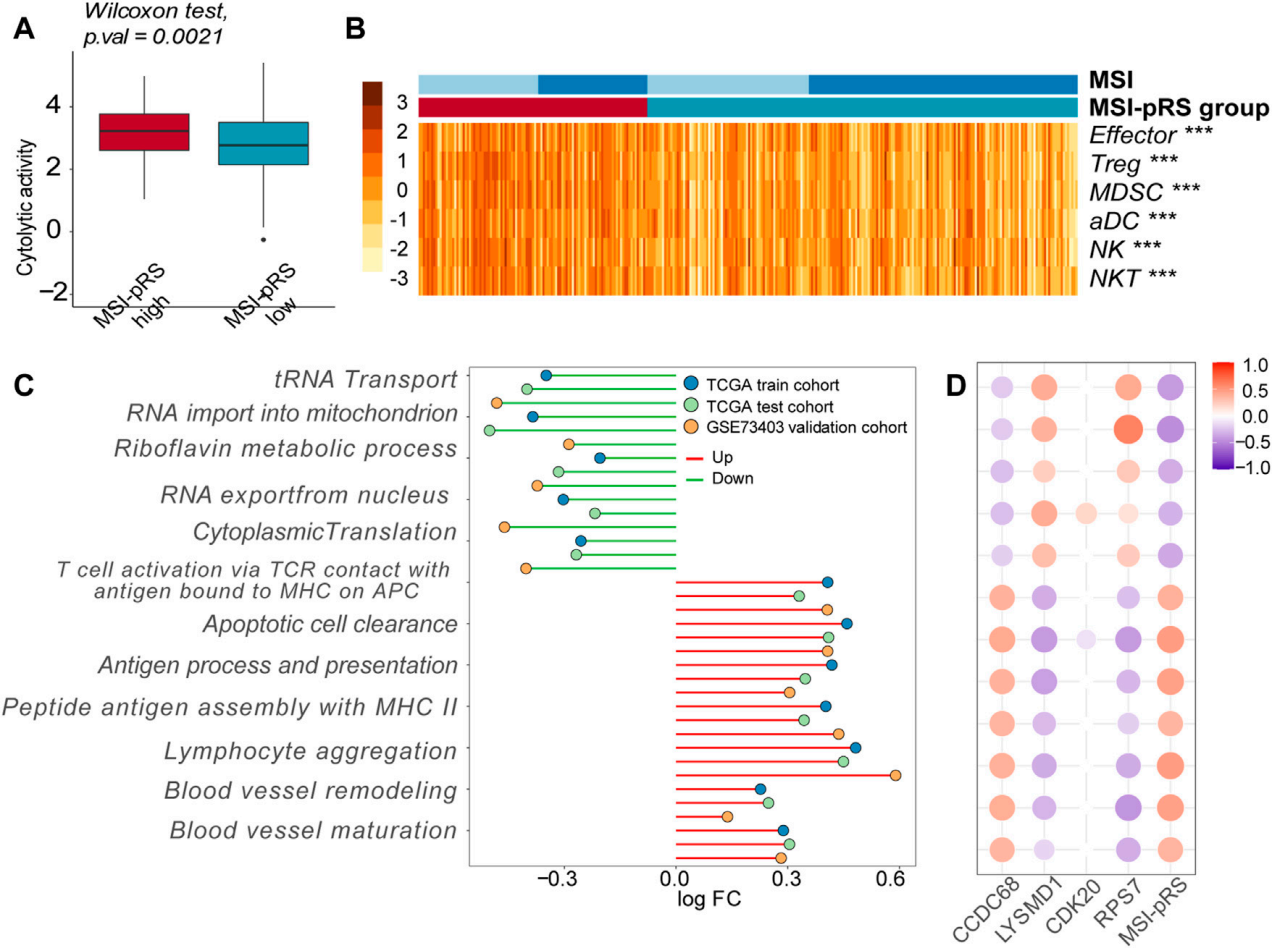
Immune features exploration of the MSI-related prognostic risk score (MSI-pRS). **(A)** Cytolytic activity (CYT) was higher in the MSI-pRS high group in the training cohort. **(B)** Heatmap of immune cells in tumor microenvironment in the training cohort. **(C)** Lollipop plot of the altered biological processes concentrating on the immune process and cell cycle, according to the MSI-pRS group in the three cohorts. **(D)** Correlation plot of the four genes in the MSI-pRS model and the altered biological processes in the TCGA training cohort. Abbreviation: LUSC, lung squamous cell carcinoma; MSI, microsatellite instability; MSI-pRS, MSI-related prognostic risk score; ICIs, immune checkpoint inhibitors; CYT, cytolytic activity; TME, tumor microenvironment; TP53, tumor protein p53; PD-L1, programmed death-ligand 1; MLH, MutL homologs; MSH, MutS homologs; TCGA, The Cancer Genome Atlas; GEO, Gene Expression Omnibus; GSEA, gene set enrichment analysis; WGCNA, weighted correlation network analysis; GO, Gene Ontology; OS, overall survival time; PFS, progression-free survival; DCA, decision curve analyses; HR, hazard ratios; ROC, receiver operating characteristic; AUC, area under the ROC curve; DEGs, differentially expressed genes; DMPs, different methylation probes; MHC, major histocompatibility complex; CCDC68, coiled-coil domain containing 68; RPS7, ribosomal protein S7; CDK20, cyclin-dependent kinase 20; and LYSMD1, LysM domain containing 1.

We then explored the immune features of the MSI-pRS group. CYT represented the ultimate anti-tumoral cytotoxicity and was higher in the MSI-pRS high group in all cohorts (Mann–Whitney’s *U*-test; *p*-value = 0.021, 0.023, 0.033; Figure 12A; Supplementary Figures S6A, B), indicating a stronger immune response in the MSI-pRS high group. Tumors with high MSI-pRS were infiltrated by a higher abundance of immune cells, including both positive immune executors and negative immune regulators in all cohorts (Figure 12B; Supplementary Figure S6C). Functional enrichment by GSVA showed that T cell activation, especially through the major histocompatibility complex (MHC) II class process, was upregulated in the MSI-pRS high group, while processes involved in protein translation were downregulated in all cohorts (Figure 12C). Moreover, blood vessel remodeling and maturation were upregulated in the MSI-pRS high group (Figure 12C). CCDC68 was positively correlated with T cell activation, while LYSMD1 and RPS7 were negatively correlated (Figure 12D, Supplementary Figure S6D).

## 4 Discussion

Lung squamous cell carcinoma comprises approximately 30% of NSCLC with a high rate of protein-altering mutations (Gerber et al., 2015). In contrast to better-known driver alterations in adenocarcinoma, such as EGFR, KRAS, and ALK, passenger mutations seem to contribute to the high somatic mutation rate in LUSC (Perez-Moreno et al., 2012). Passenger mutations dynamically accumulate under the background alternations of oncological gene drivers on the upstream of the cell cycle, such as *TP53*, for which target therapy is always invalid in LUSC. Based on the intricate genomic features of LUSC, the study explored the genome instability of LUSC and established a prognostic signature associated with it.

MSI is the molecular feature of the cancers with MMR deficiency. Ensuring high-fidelity DNA replication is essential for maintaining genome stability. The newly synthesized strand containing mismatches that have escaped proofreading by excision followed by resynthesis and ligation during DNA replication are corrected by MMR with the help of DNA replicative polymerase and DNA ligase (Hsieh and Zhang, 2017). In addition to its roles in editing replication errors, the MMR system also triggers cell cycle arrest and apoptosis in DDR. Loss of MMR results in inherited cancer susceptibility, such as Lynch syndrome, as well as an increased incidence of sporadic cancers (Knijnenburg et al., 2018; Xiao et al., 2021).

The major alternation leading to MMR-d is DNA hypermethylation in the MLH1. DNA hypermethylation or mutations (single-nucleotide polymorphism, deletion, etc) of MSH2 and other MMR genes may explain a portion of the silencing of MMR proteins and the fellow molecules. It was reported that the methylation rate of the MLH1 promoter CpG islands was 72.9% in gastric cancer and 89% in endometrial cancer. When it comes to NSCLC, the methylation rate of MLH1 was reported as 27%–35.7%, whereas the incidence of MSI-H was rare (Seng et al., 2008; Pastuszak-Lewandoska et al., 2016). The previously described phenomena indicated that epigenetical markers of MMR proteins were not consistent with MSI status and genomic instability. Generally, despite the complexity of the LUSC genome with high mutation rates, MSI is not commonplace with the incidence of less than 1% (Xiao et al., 2014; Yanagawa et al., 2021). We proposed that MSI status potentially performs its roles in genomic instability with other mechanisms.

The MSI phenotypes were defined in the LUSC by the unsupervised classification. TCGA–LUSC data were divided into MSI-H and MSI-L groups, according to MMR protein expression. The MSI status of each sample was identified by MSI score based on the expression of MMR proteins. Compared with normal samples, LUSC samples had higher MSI score, and patients with an MSI-H phenotype had higher MSI score. The MSI-H phenotype was characterized by high TMB, DNA content, and rate of aneuploidy. In addition, *TP53* was at a high rate of aberrations in the MSI-H cluster. MSI-H tumors displayed high genomic instability, which was tightly associated with the clonally expanded mutations in cancer driver genes and led to tumor heterogeneity. The MSI status defined by MMR proteins in this study revealed the severity of genomic instability in LUSC.

The MSI status was relatively clustered according to MMR proteins in the dataset and discriminated the genomic features of LUSC in some respects. The MSI-pRS defined the clinical outcome of LUSC patients based on the MSI status. The MSI-pRS was composed of CCDC68, LYSMD1, RPS7, and CDK20 and was negatively correlated with the MSI score. Compared with tumor tissues, the MSI-pRS was higher in normal tissues. The MSI-pRS of the MSI-H group was lower than that of the MSI-L group. The MSI-pRS was a negative prognostic indicator with good discrimination and calibration. Patients with low MSI-pRS tended to have an improved OS. DCA curves suggested that the model added extra value to the prognosis not only in the training cohort but also in the internal and external test cohorts.

The association of MSI-pRS and MSI status was explored. Low MSI-pRS was associated with high genomic instability with high tumor heterogeneity and TMB. The *TP53* gene encodes the p53 protein which acts as the guardian of the genome to preserve genomic integrity (Marei et al., 2021). The MSI-pRS low group had a higher incidence of *TP53* mutation and DNA hypomethylation of *TP53*. Aneuploidy is defined as the unbalanced number of chromosomes and is a salient feature of cancer genomes. Chromosome 3 alterations including 3p loss and 3q amplification participated in the tumorigenesis of LUSC. The incidence of aneuploidy was detected more frequently in the MSI-pRS low group. Moreover, LUSC with 3p loss or 3q amplification had a higher MSI-pRS. Functional enrichment declared that low MSI-pRS was associated with the upregulation of a DNA damage response signal, DNA replication initiation, cell cycle checkpoint signaling, and transcriptional and translational control. Past studies confirmed that MSI-H is associated with better survival in colorectal cancers but is inconclusive in other cancers. Low MSI-pRS was characterized by genomic instability and was associated with better clinical outcomes (Popat et al., 2005). The association of genomic stability and clinical outcomes found in the study was consistent with that of the previous study. The MSI-pRS shows promise to be an optional prognostic genomic biomarker in LUSC as the substitute of MSI.

Tumors with high MSI-pRS are displayed as an inflamed immune phenotype with acquired immune escape. Both immune effectors (active T cells and effect memorial T cells) and immune regulators (Tregs and MDSCs) were also highly infiltrated in the MSI-pRS high group. Functional enrichment indicated that processes associated with antigen presentation by MHC II were upregulated. Moreover, blood vessel remodeling and maturation might contribute to high-grade malignancy. The MSI-pRS high group was characterized by an inflamed immunophenotype, while the MSI-pRS low group was characterized by a cold one. Patients with high MSI-pRS might be the potential subpopulation that derives profit from ICIs.

The elements of MSI-pRS were explored. CCDC68 was positively correlated with MSI-pRS and is a tumor-suppressive gene by reducing cell proliferation and enhancing apoptosis, which is strongly expressed in the lung cancer tissues (Hua et al., 2020). The gene predicted short OS in LUSC patients. MSI-H, 3p loss, and 3q amp samples tended to have lower CCDC68. The gene was negatively correlated with DNA replication initiation and cell cycle checkpoint, but positively correlated with tumorigenesis-associated immune response and tumorous vessel modification. The findings declared that CCDC68 had a suppressive role in the genomic instability and a promoting role in the inflamed TME.

LYSMD1 encodes a highly conservative receptor containing a lysin motif domain, the role of which in cancerogenesis has not been elucidated so far. In the study, we first declared that LYSMD1 was a promoter of genomic instability. LYSMD1 was a positive prognostic biomarker and was negatively correlated with MSI-pRS. LUSC samples with MSI-H, *TP53* mut, 3p loss, and 3q amplification were more likely to have a higher expression of LYSMD1. The gene was positively correlated with MMR proteins and participated in active DNA replication and transcription. Nevertheless, LYSMD1 was negatively correlated with anti-cancer immunity, which might contribute to cold TME and immunosuppression.

RPS7 encodes a component of the small 40 S subunit of the ribosome, which is a critical performer in protein translation (Wu et al., 2021). The gene was a positive prognostic biomarker and negatively correlated with MSI-pRS. RPS7 is upregulated in the MSI-H, *TP53* mut, 3p loss, and 3q amplification LUSC samples. The gene also contributed to active DNA replication, transcription, and translation, especially in the activity of ubiquitin ligase in the post-translational modification. RPS7 might contribute to the cold TME with the negative correlation with immune cells in the TME.

CDK20 was once reported as a promoter of the G1/S transition and a regulator of G0/G1 checkpoint. Overexpression of CDK20 promotes proliferation and is regarded as a tumorigenesis-related factor in many cancers (Lai et al., 2020). CDK20 was a risk factor of clinical outcomes and positively correlated with MSI-pRS. The MSI-pRS was higher in the MSI-H and was positively correlated with the expression of MMR proteins, especially in the *TP53* wt group. However, *TP53* mut, 3p loss, and 3q amplification LUSC samples had no difference in CDK20 compared with the wild-type samples.

There were some limitations in the study. First, the MSI status defined in this study was a relative concept for it was classified based on the expression of MMR proteins in the TCGA–LUSC dataset. As it was declared before, MSI-H in LUSC represents a type of genetic instability. We observed the higher expression of MMR proteins in the MSI-H samples compared with the normal and MSI-L samples, which was different from that seen in colorectal cancer. The consistent trend of DNA methylation on the promoters and expression was observed in MSH3 and MSH6. MMR proteins might be activated by the genomic instability in the MSI-H group. So far, the most widely used MSI detection method was polymerase chain reaction (PCR) amplification of microsatellite markers using different panels available comprising a combination of mononucleotide and dinucleotide repeats. Tumors with instability at two or more of these markers were defined as being MSI-H, whereas those with instability at one repeat or showing no instability were defined as MSI-L tumors (Buhard et al., 2006). Evaluating the expression of the MMR proteins by immunohistochemistry (IHC) on histological tissue sections has been regarded as a valid surrogate to identify tumors with a higher probability of instability. The two methods also involved a discordance because MSI also resulted from other mechanisms (Cherri et al., 2022). The two methods are inappropriate for the cancers with low incidence of MSI and MMR dysfunction. With the widespread use of next-generation sequencing (NGS), it has been an alternative molecular test for assessing MSI and other genomic features, such as TMB and MATH, as well as revealing the molecular mechanisms leading to genomic instability. A panel of MSI-associated gene expression of tumors based on the transcriptomic data might identify the MSI status of cancer with low incidence of MSI (Li et al., 2020). Therefore, we suggested that MSI status in LUSC could be distinguished according to the expression of MMR.

Second, MSI-H tumors in the study were characterized by high DNA ploidy and aneuploidy scores, which were commonly derived from CIN. Generally, CIN represents abbreviations in DNA content and is mutually exclusive with MSI (Yoshioka et al., 2019). Both CIN and MSI result from genomic instability and derive from replication stress-associated DNA double-strand breaks (DSBs). CIN develops when DSBs are not effectively repaired by homologous recombination under the circumstance of an MMR proficient background (Matsuno et al., 2019). MSI and hypermutation generate by erroneously repaired DSBs when MMR systems are deficient concurrently. Based on the previously described processes, we assumed that MMR proteins acted as regulators of genomic instability in LUSC, which is reflected by MSI status clustered by expression of MMR proteins.

Furthermore, the MSI-pRS was constructed with an aim of excavating the meaning of MSI in LUSC profoundly. More prospective cohort studies are needed to estimate the clinical significance of the MSI-pRS signature, which might be accomplished by direct immunohistochemistry or gene sequencing of tissues. In the study, we proposed bioinformatics evidence for the correlation between the MSI-pRS and immune phenotype and inferred that LUSC patients with high MSI-pRS could derive benefits from ICIs and the combination of ICIs and angiogenetic therapy. However, the molecular mechanism needs more experimental verification.

## 5 Conclusion

In conclusion, we performed an integrative analysis to explore the MSI status in LUSC. Four hub MSI-related genes, namely, CCDC68, LYSMD1, RPS7, and CDK20, were identified and used to establish a prognostic score related to genomic instability named MSI-pRS in LUSC. Low MSI-pRS predicted better OS. LUSC with low MSI-pRS was associated with increased genomic instability and cold immunophenotype. MSI-pRS is a promising prognostic biomarker in LUSC as the substitute of MSI. In addition, we first declared the promotive role of LYSMD1 in genomic instability of LUSC. Our findings provided new insights in the biomarker finder of LUSC.

## Data Availability

The original contributions presented in the study are included in the article/Supplementary Material; further inquiries can be directed to the corresponding author.

## Supplementary material

Supplementary Figure S1 Genes in the blue, green, brown and red modules.

Supplementary Figure S2 The process of Least shrinkage and selection operator.

Supplementary Figure S3 Relationship between MSI-related prognostic risk score (MSI-pRS) and MSi status.

Supplementary Figure S4 Genomic feature exploration of MSI-related prognostic risk score (MSI-pRS).

Supplementary Figure S5 Functional enrichment in the GSE73403 external validation cohort.

Supplementary Figure S6 Immune features of MSI-pRS high and MSI-pRS low group in the TCGA-LUSC internal validation cohort and the GSE73403 external validation cohort.
