# Supplementary figures and images for: Microsatellite instability-related prognostic risk score (MSI-pRS) defines a subset of lung squamous cell carcinoma (LUSC) patients with genomic instability and poor clinical outcome
Source: Front Genet. 2023 Feb 17;14:1061002. doi: 10.3389/fgene.2023.1061002 (PMC9981642; doi:10.3389/fgene.2023.1061002)

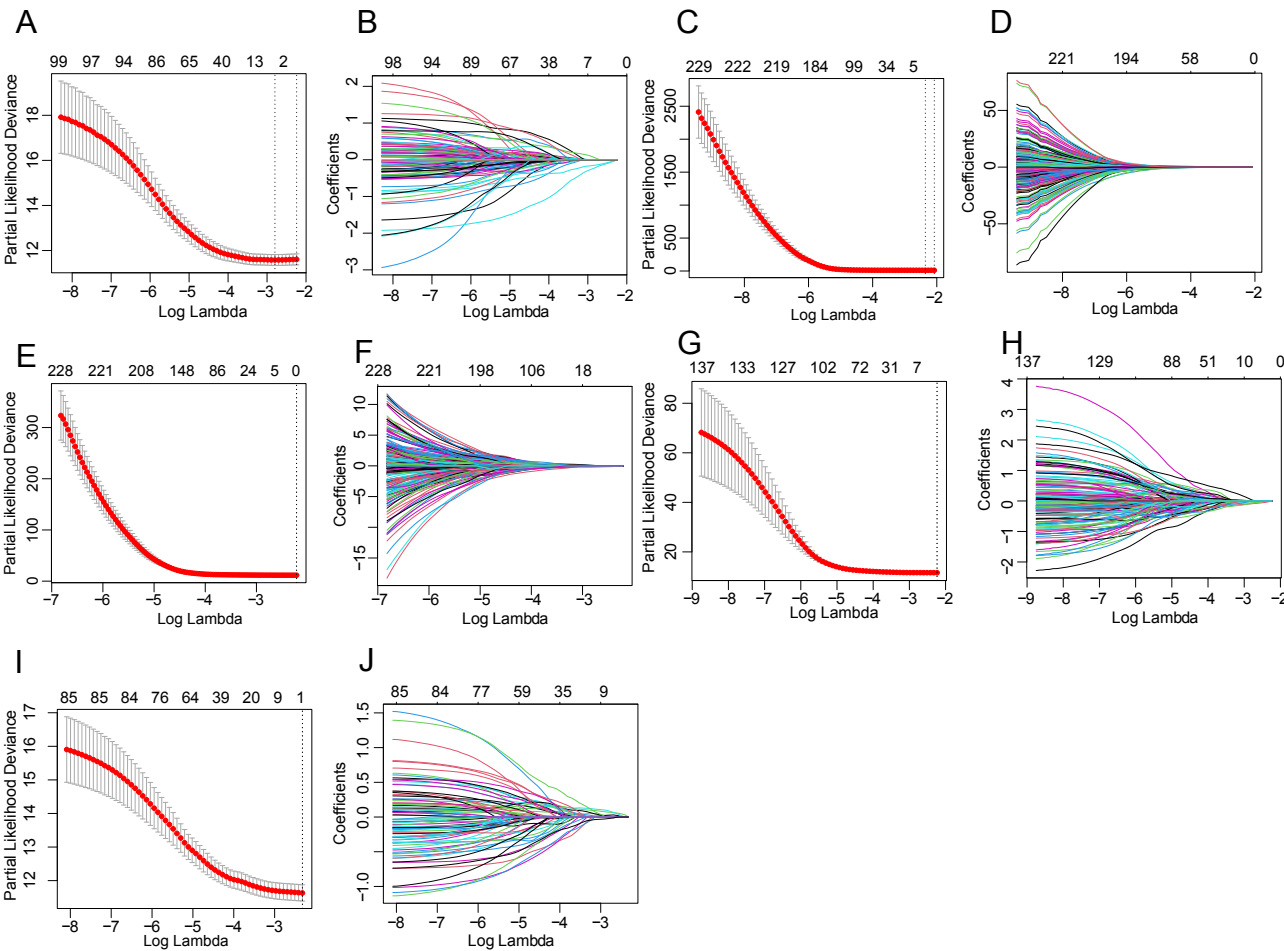

Supplement: Supplementary file 1 [file DataSheet2.PDF]

**A** *Chisq.test, p.val = 0.008*

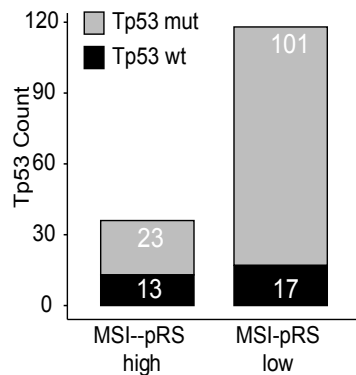

**B**

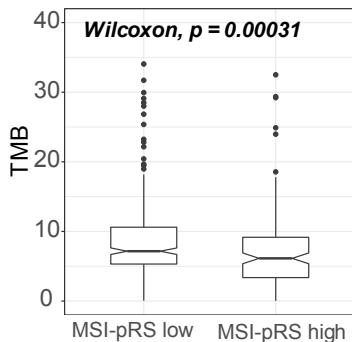

**C**

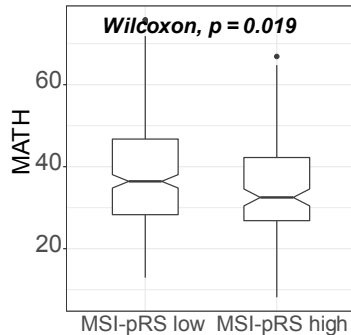

**D**

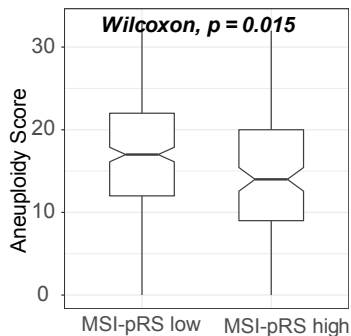

**E**

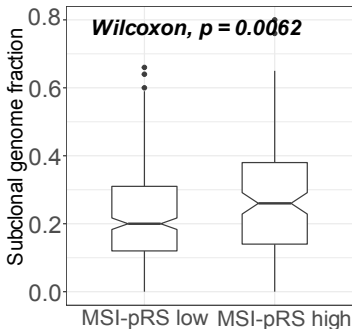

**F**

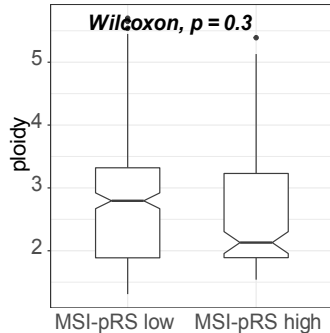

Supplement: Supplementary file 3 [file DataSheet4.PDF]

A

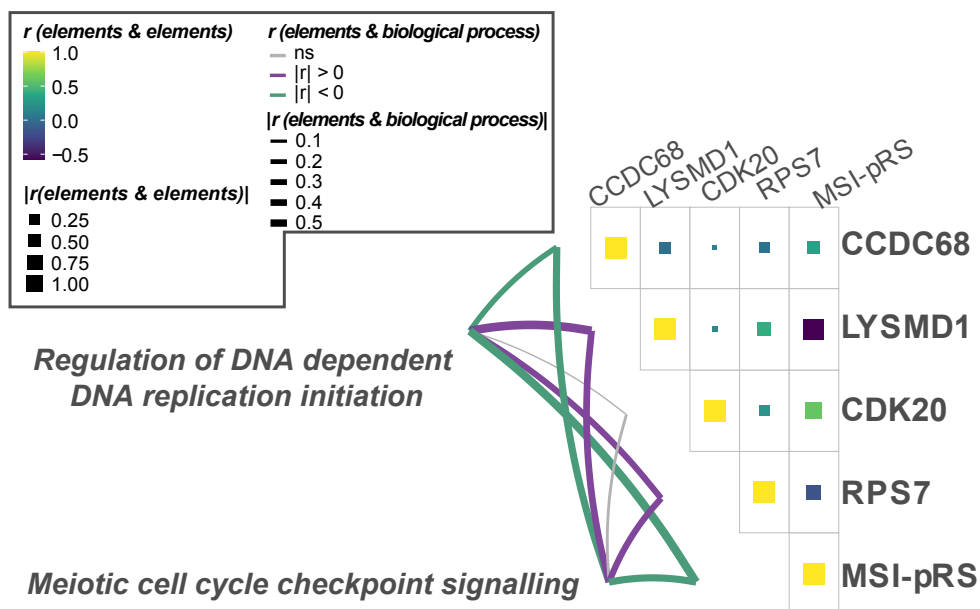

B

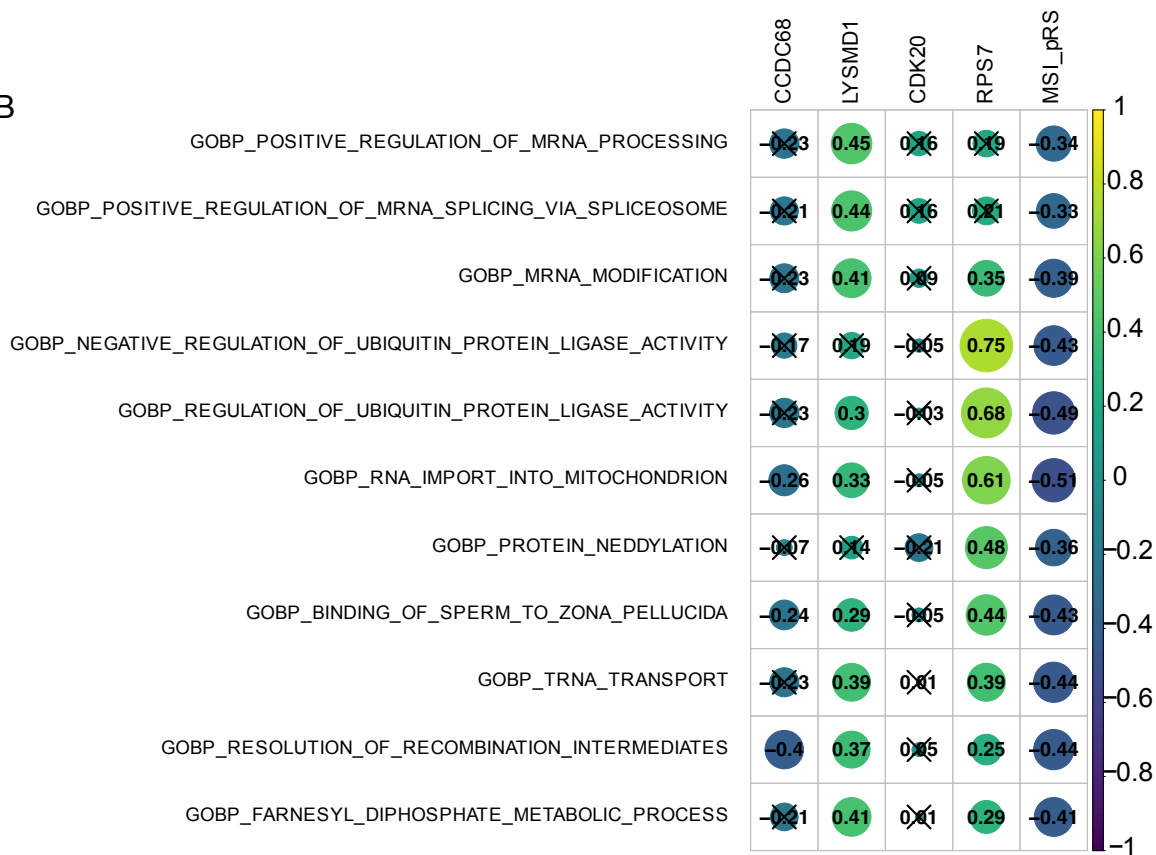

Supplement: Supplementary file 4 [file DataSheet6.PDF]

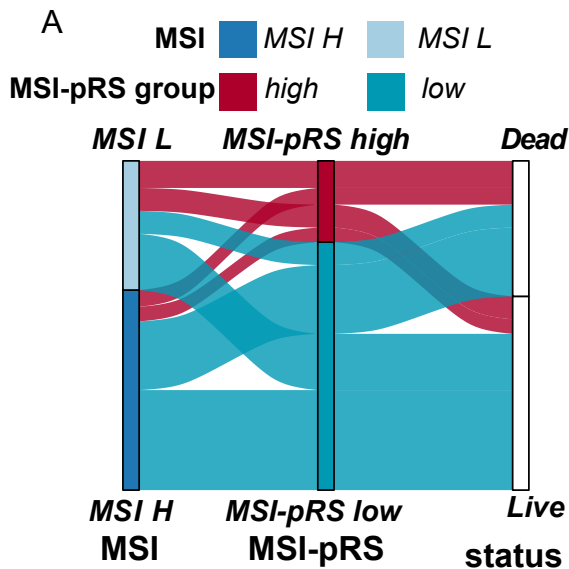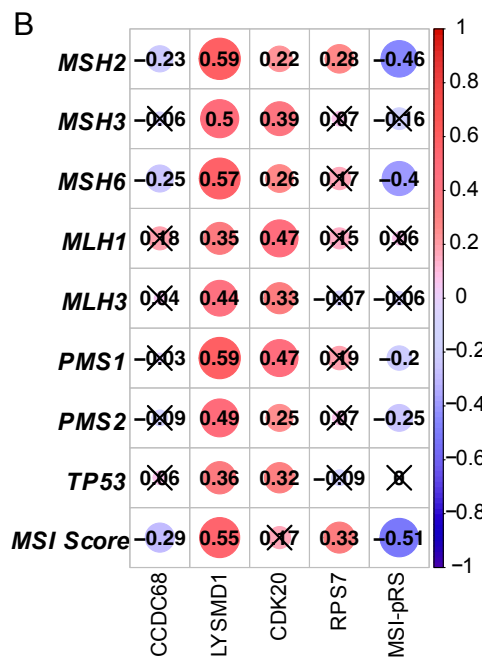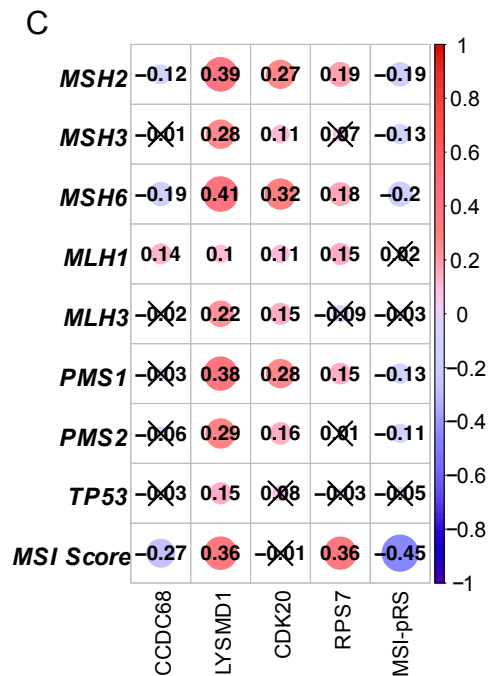

Supplement: Supplementary file 6 [file DataSheet3.PDF]
