# Supplementary material for: Microsatellite instability-related prognostic risk score (MSI-pRS) defines a subset of lung squamous cell carcinoma (LUSC) patients with genomic instability and poor clinical outcome
Source: Front Genet. 2023 Feb 17;14:1061002. doi: 10.3389/fgene.2023.1061002 (PMC9981642; doi:10.3389/fgene.2023.1061002)
Supplement: Supplementary file 5 [file Table1.DOCX]

***Table*** ***S1:*** *Summary descriptives table by groups of MSI status of TCGA LUSC cohort*

|  | **[ALL]** | ***MSI-low*** | ***MSI-high*** | **P.val** |
| --- | --- | --- | --- | --- |
|  | ***N=472*** | ***N=196*** | ***N=276*** |  |
| **Age** | 67.2 (8.51) | 67.5 (8.53) | 66.9 (8.50) | 0.439 |
| **Gender** |  |  |  | 0.928 |
| female | 123 (26.1%) | 52 (26.5%) | 71 (25.7%) |  |
| male | 349 (73.9%) | 144 (73.5%) | 205 (74.3%) |  |
| **Stage** |  |  |  | 0.220 |
| Stage I | 229 (48.9%) | 101 (51.8%) | 128 (46.9%) |  |
| Stage II | 151 (32.3%) | 60 (30.8%) | 91 (33.3%) |  |
| Stage III | 81 (17.3%) | 29 (14.9%) | 52 (19.0%) |  |
| Stage IV | 7 (1.50%) | 5 (2.56%) | 2 (0.73%) |  |
| **Smoking history**  **(pack years)** | 52.7 (29.6) | 53.4 (32.7) | 52.2 (27.1) | 0.686 |
