# Supplementary material for: Microsatellite instability-related prognostic risk score (MSI-pRS) defines a subset of lung squamous cell carcinoma (LUSC) patients with genomic instability and poor clinical outcome
Source: Front Genet. 2023 Feb 17;14:1061002. doi: 10.3389/fgene.2023.1061002 (PMC9981642; doi:10.3389/fgene.2023.1061002)
Supplement: Supplementary file 7 [file DataSheet1.PDF]

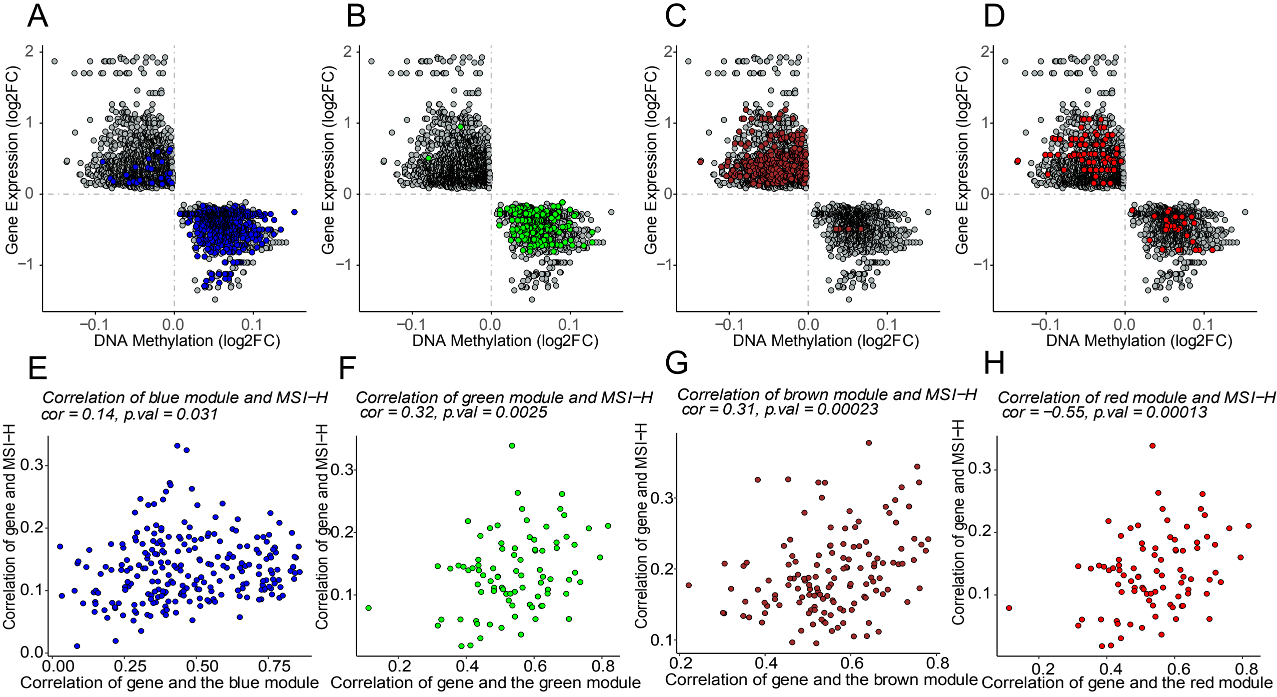
A
B
C
D
Gene Expression (log2FC)
DNA Methylation (log2FC)
E
Correlation of blue module and MSI−H
cor = 0.14, p.val = 0.031
Correlation of gene and MSI−H
Correlation of gene and the blue module
F
Correlation of green module and MSI−H
cor = 0.32, p.val = 0.0025
Correlation of gene and the green module
G
Correlation of brown module and MSI−H
cor = 0.31, p.val = 0.00023
Correlation of gene and the brown module
H
Correlation of red module and MSI−H
cor = −0.55, p.val = 0.00013
Correlation of gene and the red module
