# Supplementary material for: Microsatellite instability-related prognostic risk score (MSI-pRS) defines a subset of lung squamous cell carcinoma (LUSC) patients with genomic instability and poor clinical outcome
Source: Front Genet. 2023 Feb 17;14:1061002. doi: 10.3389/fgene.2023.1061002 (PMC9981642; doi:10.3389/fgene.2023.1061002)
Supplement: Supplementary file 8 [file DataSheet5.PDF]

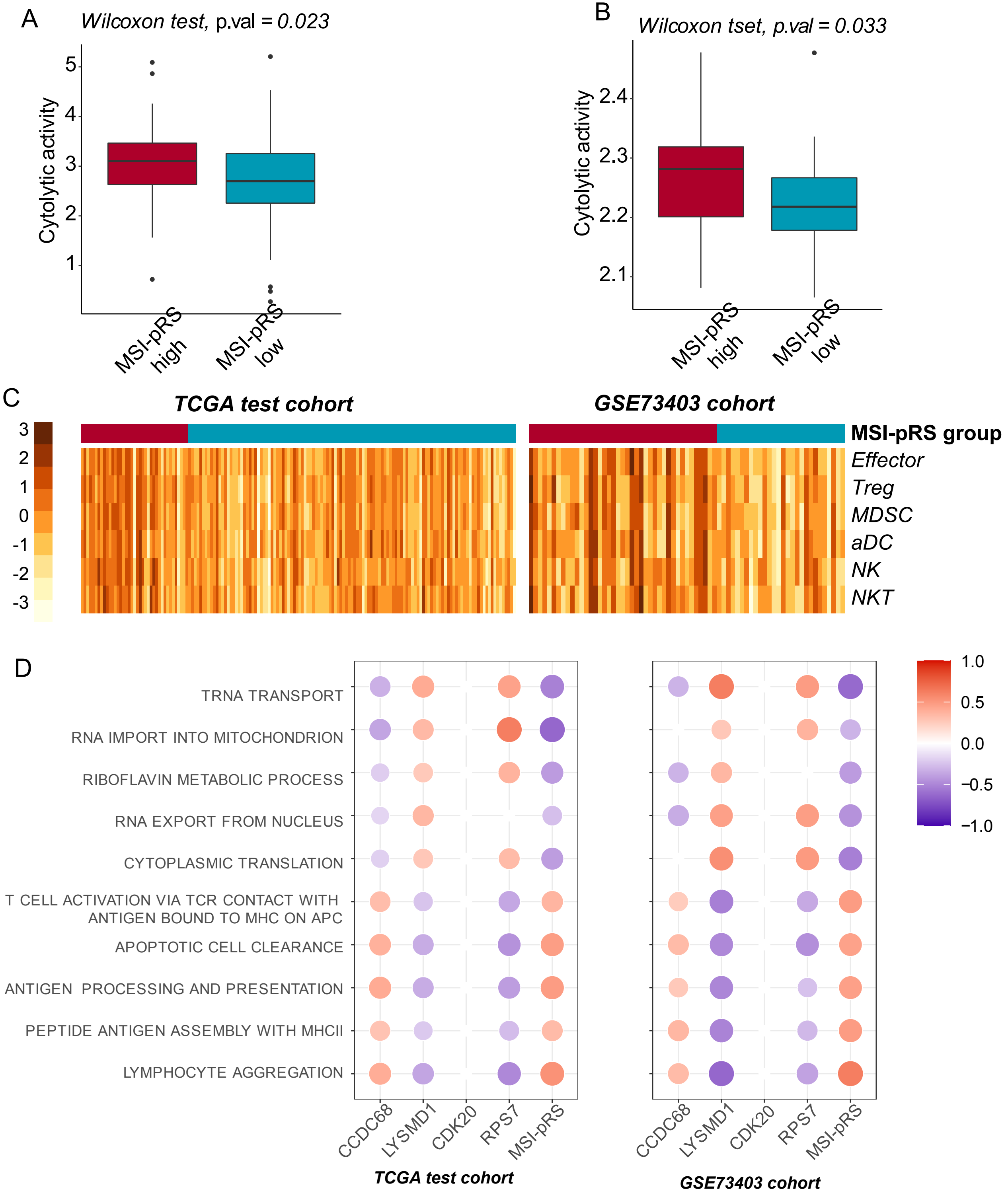
A
Wilcoxon test, p.val = 0.023
Cytolytic activity
MSI-pRS high
MSI-pRS low
B
Wilcoxon tset, p.val = 0.033
Cytolytic activity
MSI-pRS high
MSI-pRS low
C
TCGA test cohort
GSE73403 cohort
MSI-pRS group
Effector
Treg
MDSC
aDC
NK
NKT
D
TRNA TRANSPORT
RNA IMPORT INTO MITOCHONDRION
RIBOFLAVIN METABOLIC PROCESS
RNA EXPORT FROM NUCLEUS
CYTOPLASMIC TRANSLATION
T CELL ACTIVATION VIA TCR CONTACT WITH ANTIGEN BOUND TO MHC ON APC
APOPTOTIC CELL CLEARANCE
ANTIGEN PROCESSING AND PRESENTATION
PEPTIDE ANTIGEN ASSEMBLY WITH MHCII
LYMPHOCYTE AGGREGATION
CCDC68
LYSMD1
CDK20
RPS7
MSI-pRS
TCGA test cohort
GSE73403 cohort
