# Supplementary material for: Microsatellite instability-related prognostic risk score (MSI-pRS) defines a subset of lung squamous cell carcinoma (LUSC) patients with genomic instability and poor clinical outcome
Source: Front Genet. 2023 Feb 17;14:1061002. doi: 10.3389/fgene.2023.1061002 (PMC9981642; doi:10.3389/fgene.2023.1061002)
Supplement: Supplementary file 9 [file Table3.DOCX]

**Table S6-1**: Summary descriptives table by MSI-pRS group of TCGA training cohort

|  | **[ALL]** | ***MSI-pRS high*** | ***MSI-pRS low*** | **P.val** |
| --- | --- | --- | --- | --- |
|  | ***N=314*** | ***N=109*** | ***N=205*** |  |
| **Age** | 67.3 (8.48) | 68.2 (8.74) | 66.8 (8.32) | 0.193 |
| **Gender** |  |  |  | 1.000 |
| female | 76 (24.2%) | 26 (23.9%) | 50 (24.4%) |  |
| male | 238 (75.8%) | 83 (76.1%) | 155 (75.6%) |  |
| **Stage** |  |  |  | 0.943 |
| StageI-II | 255 (81.7%) | 89 (82.4%) | 166 (81.4%) |  |
| StageIII-IV | 57 (18.3%) | 19 (17.6%) | 38 (18.6%) |  |
| **Smoking history**  **(pack years)** | 53.1 (30.2) | 53.5 (30.1) | 53.0 (30.4) | 0.898 |
| **MSI-pRS** | -2.13 (0.41) | -1.68 (0.23) | -2.36 (0.27) | <0.001 |

***Table S6-2:*** Summary descriptives table by MSI-pRS group of TCGA validation cohort

|  | ***[ALL]*** | ***MSI-pRS high*** | ***MSI-pRS low*** | **P.val** |
| --- | --- | --- | --- | --- |
|  | ***N=158*** | ***N=39*** | ***N=119*** |  |
| **Age** | 66.9 (8.60) | 69.1 (9.14) | 66.2 (8.33) | 0.090 |
| **Gender** |  |  |  | 0.048 |
| female | 47 (29.7%) | 17 (43.6%) | 30 (25.2%) |  |
| male | 111 (70.3%) | 22 (56.4%) | 89 (74.8%) |  |
| **Stage** |  |  |  | 0.132 |
| StageI-II | 125 (80.1%) | 35 (89.7%) | 90 (76.9%) |  |
| StageIII-IV | 31 (19.9%) | 4 (10.3%) | 27 (23.1%) |  |
| **Smoking history**  **(pack years)** | 51.9 (28.3) | 49.3 (27.9) | 52.7 (28.5) | 0.553 |
| **MSI-pRS** | -2.09 (0.41) | -1.58 (0.24) | -2.26 (0.29) | <0.001 |

***Table S3-3:*** Summary descriptives table by MSI-pRS group of GSE73403 validation cohort

|  | | ***[ALL]*** | ***MSI-pRS high*** | ***MSI-pRS low*** | **P.val** |
| --- | --- | --- | --- | --- | --- |
|  | | ***N=69*** | ***N=41*** | ***N=28*** |  |
| **Age** | 59.6 (9.33) | | 60.1 (9.42) | 58.7 (9.29) | 0.534 |
| **Gender** |  | |  |  | 0.641 |
| Female | 4 (5.80%) | | 3 (7.32%) | 1 (3.57%) |  |
| Male | 65 (94.2%) | | 38 (92.7%) | 27 (96.4%) |  |
| **Stage** |  | |  |  | 0.665 |
| StageI-II | 46 (66.7%) | | 26 (63.4%) | 20 (71.4%) |  |
| StageIII-IV | 23 (33.3%) | | 15 (36.6%) | 8 (28.6%) |  |
| **Smoking history**  **(pack years)** | 33.5 (26.0) | | 33.0 (28.1) | 34.2 (23.1) | 0.846 |
| **MSI-pRS** | -3.46 (0.43) | | -3.18 (0.27) | -3.88 (0.24) | <0.001 |
