# Supplementary material for: Microsatellite instability-related prognostic risk score (MSI-pRS) defines a subset of lung squamous cell carcinoma (LUSC) patients with genomic instability and poor clinical outcome
Source: Front Genet. 2023 Feb 17;14:1061002. doi: 10.3389/fgene.2023.1061002 (PMC9981642; doi:10.3389/fgene.2023.1061002)
Supplement: Supplementary file 10 [file Table4.DOCX]

TableS7-1 Subgroup analysis of MSI-pRS in the TCGA training cohort

| Clinic characteristics | Patient number | Hazard ratio | 95%CI (lower) | 95%CI (upper) | P-value |
| --- | --- | --- | --- | --- | --- |
| Gender |  |  |  |  |  |
| Female | 76 | 2.73 | 1.13 | 6.63 | 0.026 |
| Male | 238 | 2.79 | 1.77 | 4.42 | <0.001 |
| Age group |  |  |  |  |  |
| < 65 | 208 | 2.6 | 1.6 | 4.22 | <0.001 |
| >= 65 | 110 | 2.9 | 1.41 | 5.96 | 0.004 |
| Stage |  |  |  |  |  |
| StageI-II | 257 | 2.78 | 1.73 | 4.47 | <0.001 |
| StageIII-IV | 59 | 2.35 | 1.08 | 5.1 | 0.03 |
| Lymphy node stage |  |  |  |  |  |
| Negative | 192 | 2.14 | 1.24 | 3.68 | 0.006 |
| Positive | 128 | 3.65 | 1.92 | 6.91 | <0.001 |
| Smoking history |  |  |  |  |  |
| Heavy | 270 | 2.68 | 1.7 | 4.2 | <0.001 |
| Ordinary | 91 | 2.41 | 0.53 | 10.85 | 0.252 |
| MSI |  |  |  |  |  |
| MSI-L | 134 | 3.04 | 1.71 | 5.39 | <0.001 |
| MSI-H | 180 | 2.49 | 1.33 | 4.68 | 0.004 |
|  |  |  |  |  |  |

TableS7-2 Subgroup analysis of MSI-pRS in the TCGA test cohort

| Clinic characteristics | Patient number | Hazard ratio | 95%CI (lower) | 95%CI (upper) | P-value |
| --- | --- | --- | --- | --- | --- |
| Gender |  |  |  |  |  |
| Female | 47 | 1.1 | 0.27 | 4.54 | 0.893 |
| Male | 111 | 2.19 | 1.13 | 4.24 | 0.02 |
| Age group |  |  |  |  |  |
| < 65 | 102 | 1.84 | 0.91 | 3.74 | 0.091 |
| >= 65 | 57 | 1.87 | 0.48 | 7.27 | 0.366 |
| Stage |  |  |  |  |  |
| StageI-II | 127 | 2.75 | 1.38 | 5.48 | 0.004 |
| StageIII-IV | 33 | 0.64 | 0.14 | 3.01 | 0.572 |
| Lymphy node stage |  |  |  |  |  |
| Negative | 113 | 2.55 | 1.26 | 5.16 | 0.009 |
| Positive | 45 | 0.82 | 0.22 | 3 | 0.761 |
| Smoking history |  |  |  |  |  |
| Heavy | 135 | 1.8 | 0.82 | 3.94 | 0.14 |
| Ordinary | 46 | 2.26 | 0.66 | 7.77 | 0.195 |
| MSI |  |  |  |  |  |
| MSI-L | 62 | 2.16 | 0.9 | 5.2 | 0.086 |
| MSI-H | 96 | 1.55 | 0.62 | 3.84 | 0.347 |

TableS7-3 Subgroup analysis of MSI-pRS in the GSE73403 validation external test cohort

| Clinic characteristics | Patient number | Hazard ratio | 95%CI (lower) | 95%CI (upper) | P-value |
| --- | --- | --- | --- | --- | --- |
| Gender |  |  |  |  |  |
| Female | 4 | 8.97906E+12 | 0 | 1.19E+41 | 0.367 |
| Male | 65 | 2.51 | 1.02 | 6.15 | 0.044 |
| Age group |  |  |  |  |  |
| < 65 | 44 | 1.89 | 0.54 | 6.65 | 0.318 |
| >= 65 | 25 | 3.3 | 0.84 | 12.9 | 0.086 |
| Stage |  |  |  |  |  |
| StageI-II | 46 | 7.41 | 1.37 | 40.04 | 0.02 |
| StageIII-IV | 23 | 1.23 | 0.4 | 3.83 | 0.715 |
| Lymphy node stage |  |  |  |  |  |
| Negative | 35 | 5 | 0.79 | 31.47 | 0.087 |
| Positive | 34 | 1.93 | 0.67 | 5.55 | 0.223 |
| Smoking history |  |  |  |  |  |
| Heavy | 46 | 3.1 | 1.2 | 8.03 | 0.02 |
| Ordinary | 23 | 1.4 | 0.2 | 9.9 | 0.736 |
| MSI |  |  |  |  |  |
| MSI-L | 38 | 1.5 | 0.31 | 7.34 | 0.614 |
| MSI-H | 31 | 4.76 | 1.22 | 18.55 | 0.025 |
